# Macrophage KDM2A promotes atherosclerosis via regulating FYN and inducing inflammatory response

**DOI:** 10.7150/ijbs.102675

**Published:** 2025-03-31

**Authors:** Yuzhou Xue, Yuce Peng, Ling Jin, Lin Liu, Qian Liu, Xiaofan Yuan, Jingyu Wang, Mingming Zhao, Wenming Zhang, Suxin Luo, Yuanjing Li, Minghao Luo, Longxiang Huang

**Affiliations:** 1 Department of Cardiology and Institute of Vascular Medicine, NHC Key Laboratory of Cardiovascular Molecular Biology and Regulatory Peptides, State Key Laboratory of Vascular Homeostasis and Remodeling, Peking University Third Hospital, Beijing, China.; 2 Department of Cardiovascular Medicine, Cardiovascular Research Center, The First Affiliated Hospital of Chongqing Medical University, Chongqing, China.; 3 Department of Dermatology, Beijing Hospital, National Center of Gerontology, Institute of Geriatric Medicine, Chinese Academy of Medical Sciences & Peking Union Medical College, Beijing, China.; 4 College of Laboratory Medicine, Chongqing Medical University, Chongqing, China.; 5 General Practice, Sichuan Provincial People's Hospital, School of Medicine, University of Electronic Science and Technology of China, Chengdu, China.; 6 Renal Division, Peking University First Hospital, Beijing, China.; Yuzhou Xue, Yuce Peng, and Ling Jin contributed equally in this paper.

**Keywords:** Atherosclerosis, KDM2A, macrophage, oxidative stress, inflammatory response, FYN, Metabolism reprograming.

## Abstract

Macrophage inflammatory response is the key driver in atherosclerosis development. However, transcriptional remodeling of macrophage inflammatory response remains largely unknown. In this study, transcriptional regulatory networks were constructed from human plaque microarray datasets. Differential analysis and subsequent machine learning algorithms were used to identify key transcriptional regulons. Multiple immune cell inference methods (including CIBERSORT, ssGSEA, MCP-counter, and xCell), single-cell RNA-seq of human plaques and immunofluorescence of human and mouse plaque samples reveal that the macrophage-specific transcriptional regulator, KDM2A, is critical for inflammatory response. Diagnostic analyses validate KDM2A expression in peripheral monocytes/macrophages is an excellent predictor of atherosclerosis development and progression. RNA-seq of mouse bone marrow-derived macrophages under oxidized low-density lipoprotein stimulation reveal KDM2A knockdown significantly represses pro-inflammatory, oxidative, and lipid uptake pathways. In vitro experiments confirmed KDM2A activates inflammation, oxidative stress and lipid accumulation in macrophages. Mechanistically, FYN was identified as a direct target of KDM2A by chromatin immunoprecipitation followed by sequencing and qPCR analysis. Specific inhibition of FYN restored the inflammatory response, oxidative stress, and intracellular lipid accumulation after transfection with KDM2A overexpression plasmid. Importantly, macrophage-specific knockdown of KDM2A in ApoE^-/-^ mice fed a high-fat diet apparently attenuated plaque progression. Furthermore, the genetic association of KDM2A with atherosclerosis was validated by Mendelian randomization and colocalization analysis. A group of small molecules with the potential to target KDM2A has been identified through virtual screening, offering promising strategies for atherosclerosis treatment. The current study provides the novel role of KDM2A in macrophage inflammatory response of atherosclerosis through transcriptional regulation of FYN.

## Introduction

Atherosclerosis is the leading cause of cardiovascular disease, and its complications, including myocardial infarction and stroke, are the first and fifth causes of death from this disease, respectively 1. During atherogenesis, a variety of cell types, including monocytes/macrophages, vascular smooth muscle cells (VSMCs), and endothelial cells (ECs), undergo fate conversion to multipotential cells that can adopt plaque-destabilizing (inflammation, calcification) or plaque-stabilizing (collagen deposition, resident-like) cell states 2-4. The imbalanced or dysregulated phenotypic transformation of cells is the prerequisite for atherogenesis. For example, macrophage inflammation and subsequent foamy cell formation are the major driver of atherosclerosis development 5-7. Macrophages in the subendothelial space undergo phenotypic alterations upon oxidized low-density lipoprotein (ox-LDL) uptake, leading to lipid dysregulation and thereafter cytokines release 8. With the development of single-cell multi-omics technology, more specific characteristics of different macrophage phenotypes have been delineated, including inflammatory, resident-like, foamy/TREM2, IFNIC, and cavity macrophages 9-11. Accordingly, the emerging role of transcriptional remodeling in modulation of cell phenotype has attracted attention 12.

In the work of Samuel and his colleagues, approximately 1,600 human transcriptional factors (TFs) were systematically summarized and defined according to the presence of a DNA binding domain or literature information (because of the broader connotation, hereafter referred to as transcriptional regulators) 13. Recently, several studies have suggested that newly identified transcriptional regulators (TRs) (such as NFATc3, ARID3A, CREB, and etc.) play an increasingly important role in macrophage activation and the development of atherosclerosis 14-16. However, no study to date has systematically shown how TRs and their potential interactions induce the development of atherosclerosis and potential phenotypic transition of macrophage. Here, we applied a novel pipeline to unbiased screening key TRs that play a critical role in the development of atherosclerosis. And, lysine demethylase 2A (KDM2A) was identified as a key TR that specifically induces macrophage inflammatory response and atherosclerosis development through high-throughput data and in vivo experiments.

KDM2A has been reported to demethylate the di-methylated H3K36 residue to transcriptionally regulate gene expression 17. Previous studies have shown that KDM2A is associated with tumorigenesis, progression, poor prognosis of various cancers (esophageal squamous cell carcinoma, lung cancer, breast cancer, etc.), and obesity 18-22. In Longmin's work, they found that loss of Kdm2a promotes Pparg transcription by inhibiting its H3K36me2 demethylation, thereby protecting against from high fat diet-induced obesity.^18^ In addition, KDM2A can promote the de-clustering of alternative telomere elongation to sustain the replicative immortality of tumor cells 23. A recent study revealed that KDM2A paralog specifically interacts with nucleosome acidic patch and facilitates dynamic nucleosomal DNA unwrapping and histone charge shielding that induce the H3K36 sequence for demethylation in tumor cells 24. Although, these studies suggest that KDM2A is an important TR, no study has revealed a potential association of KDM2A in cardiovascular disease.

In this study, we screened KDM2A as a novel key TR playing critical roles in the development of atherosclerosis. It specifically regulates macrophage inflammatory response (inflammation, oxidative stress, apoptosis, and lipid accumulation) through directly transcriptional activation of FYN.

## Materials and Methods

### Experimental design

The objectives were accomplished in four steps: i) analysis and validation of the master transcriptional regulons based on transcriptome datasets from patients with atherosclerosis, ii) identification the role of the *de novo* regulon KDM2A in phenotypic alterations of macrophages, iii) follow-up *in vitro* experiments on KDM2A and its target FYN in macrophage inflammatory response, iv) validation of the atherogenic role of macrophage KDM2A by *in vivo* experiments.

### Data collection

Four independent transcriptome datasets (GSE43292, GSE40231, GSE21545, and GSE28829) comprising 299 patient-derived arterial samples were obtained from the Gene Expression Omnibus (GEO) database (detail information shown in Supplementary table 1). First, the samples were preprocessed using the affy and affyPLM packages prior to analysis 25. Briefly, probe-set summaries were calculated from the raw data (CEL files) through probe-level robust regressions and then normalized using the robust multiarray averaging (RMA) algorithm. Accounting for the same platform of GSE40231, GSE21545, and GSE28829 (a total of 235 samples), we integrated these three datasets as we previously described (called combine dataset) 26. Then, GSE43292 for uncovering regulons and the combine dataset for validation were used in the key regulon identification analysis. Utilizing the plaque classification data (early and advanced atherosclerotic plaques) available in GSE28829, we further analyzed the expression of KDM2A to investigate its involvement in atherosclerosis progression.

The microarray expression datasets (GSE90074 and GSE56045) with corresponding clinical information were also been downloaded from the GEO database for clinical significance analysis. GSE90074 include peripheral mononuclear samples from 143 subjects with coronary artery disease (CAD) classification information, while GSE56045 included 1202 peripheral monocyte/macrophage (CD14+) samples from participants in the Multi-Ethnic Study of Atherosclerosis (MESA). In addition, Genotype-Tissue Expression (GTEx) was used for correlation analysis of KDM2A and its potential targets among different organs.

### Patient sample collection

Carotid artery samples were obtained from patients who were certified for carotid endarterectomy (CEA) certification and underwent surgery at the First Affiliated Hospital of Chongqing Medical University; sampling was approved by the patient and the Ethics Committee of the First Affiliated Hospital of Chongqing Medical University (approval number 2021-689) in strict accordance with the Declaration of Helsinki on Biomedical Research Involving Human Subjects.

Blood samples were consecutively collected from patients with stable CAD who undergoing percutaneous coronary intervention at the First Affiliated Hospital of Chongqing Medical University upon admission (ethics approval no. 2019-148). Peripheral blood mononuclear cells (PBMCs) were isolated via gradient centrifugation using Ficoll-Paque PLUS density gradient media (Cytiva, 17144002). A total of 10 patients were enrolled to validate the upregulation of KDM2A, p-FYN/FYN, and associated pathways in the severe CAD group, classified based on the Gensini score as previously described 27. The TNF-α, IL-6, and HMGB concentration in the patients' serum using ELISA kits (Beyotime, Shanghai, China) as per the manufacturer's instructions. All patients had provided informed consent in our study.

### Master regulon identification

The master TRs and their regulated target genes (named as regulons) were screened using a transcriptional regulatory network construction method 28. A total of 1639 proteins identified as TRs based on their DNA-binding specificities and the regulatory effect on transcription were included to infer the transcriptional regulatory networks based on Samuel's work 13.

Using the preprocessed data, transcriptional regulatory regulons were generated and their activity assessed for further selection by RTN package. In brief, the mutual information of candidate TRs with all potential target genes was analyzed, and the non-significant and unstable associations were excluded by permutation analysis and bootstrapping. The networks were then pruned by ARACNe algorithm. Two-tailed gene set enrichment analysis (GSEA-2T) was used to test for positive or negative association with atherosclerosis.

Based on the activities of transcriptional networks, we performed differential analysis to find significantly altered regulons (P adj < 0.05 and |Fold change| > 2) by limma package 29. Then, two different techniques for feature selection were employed to identify candidate master regulons: Least Absolute Shrinkage and Selection Operator (LASSO) and Support Vector Machine Recursive Feature Elimination (SVM-RFE). The LASSO is a regularization method used in linear regression that shrinks the coefficient estimates towards zero, and the minimum criteria with 10-fold cross-validation were chosen for regulon selection by the glmnet package. SVM-RFE trains a subset of features to reduce the feature set and identify the most predictive features. The set of features with minimum 10-fold cross-validation errors were picked up by the e1071 package 30. The intersections by LASSO and SVM-RFE were recognized as potential master regulons and the difference in activity of these candidate regulons was examined in the combine dataset.

To assess the clinical significance of the potential regulons, we also performed correlation and regression analysis of potential TRs with CAD severity in GSE90074, which includes the demographic (age, sex, diabetes, hypertension, and hyperlipidemia) and pathological information (degrees of coronary artery stenosis from a geriatric cohort (phase 2 of the Supporting a Multi-disciplinary Approach to Researching Atherosclerosis study). The standards used to quantify the severity of CAD (0 to 4, with 0-1 defined as non-obstructive and ≥2 defined as obstructive) were consistent with corresponding publication 31. For the ROC analysis, we used the roc function from the pROC package (v1.18.5) and plotted the ROC curves using ggplot2. For regression analysis, we used the glm function from the stats package (v4.4.1). Correlation analyses were also performed in GSE56045 to validate the significant association of target TRs expression in monocytes/macrophages with coronary artery calcification (CAC, Agatston score) and carotid plaque score 32. In addition, the corresponding methylome data (GSE56046) were also obtained to investigate potential correlation between TR and the CpG sites of its targets.

### Functional enrichment and immune cells infiltration

GSEA was carried out to identify potentially involved pathways according to the correlation coefficients of genes with assigned regulons. GSEA was performed against multiple ontologies, namely Gene ontology (GO) terms (including molecular function, cellular component, and biological process), Kyoto Encyclopedia of Genes and Genomes (KEGG), and reference HALLMARK reference gene sets from the MSigDB database 33.

Single sample GSEA (ssGSEA) was developed to measure the proportions of 28 immune cell types. And the relative abundance of each cell type was normalized from 0 to 1 enrichment score 34. CIBERSORT can deconvolute the gene expression profile into relative percentages of 22 distinct leukocyte subsets 34. Furthermore, the Microenvironment Cell Population-counter (MCP-counter) (can quantify the absolute abundance of 8 immune and 2 stromal cell populations) and the xCell algorithm (can infer 64 immune and stromal cell types) were used to validate the immune infiltration analysis 35, 36. We systematically evaluated the immune cell abundances between normal and atherosclerotic arterial samples and their correlations with regulon activities. The consistency of associations between a leukocyte type and an assigned regulon was examined among different algorithms and independent datasets.

### scRNA-seq processing

Single-cell RNA-sequencing (scRNA-seq) (10X Genomics, GSE159677) of the proximal adjacent (PA) and atherosclerotic core (AC) portions of plaques from 3 patients was downloaded from the GEO database (Supplementary table 1) 37. And, the details of scRNA-seq data preprocessing, sample integration, cell type annotation, sub-clustering analysis, and TR activity inference were described in our previous study 38.

Pseudotime trajectory analysis was then conducted in macrophage clusters to identify different cell states and branches by monocle v2.16.0 R package. Genes defining the trajectory were selected based on *VariableFeatures* function after SCTransform. And dimensional reduction was carried out using *DDRTree* method with max components as 2. Cells expressing resident specific markers such as FOLR2, F13A1, and GAS6 were recognized as the root, as we hypothesized that atherosclerosis is an inflammation-driven disease. Genes significantly associated with the first branch point (q value < 10^-4^) were clustered through *plot_genes_branched_heatmap* function (num_clusters = 3). Gene clusters upregulated in different cell states (state 2 and 3) were functionally mapped (KEGG annotation). KDM2A activity was evaluated in these three different macrophage subtypes and states. And the correlation between KDM2A activity and pseudotime was fitted by regression model using *ggscatterstats* function of ggstatsplot package (v0.12.0).

In addition to 10X scRNA-seq dataset, GSE260657 based on SmartSeq2 (~8000 genes/cell) from 7690 single cells isolated from humans with asymptomatic (n=7) and symptomatic (n=8) carotid plaques were enrolled for validation (Supplementary table 1). Cells with a total RNA count ≤ 50,000 or ≥ 750,000, highly relative mitochondrial or ERCC gene content (≥10%), and genes expressed in ≤ 3 cells or with a total read count < 300 were excluded according to corresponding study 39. Using the *RunHarmony* function of Harmony package (v1.2.1), we integrated different samples based on *patient_ID* and performed SCT normalization before conducting t-SNE clustering. Cell types were annotated based on the highly expressed genes in each cluster. Cells identified as Myeloid cells were enrolled for the second round of integration, normalization, and dimensionality reduction analysis.

### KDM2A genetic validation

Arterial tissue-specific cis-expression quantitative trait loci (cis-eQTL) of KDM2A were integrated from the GTEx project (v8; https://gtexportal.org/home/), and those eQTLs with statistical significance (P < 1×10^-5^) were included in the subsequent analysis. Genome-Wide Association Study (GWAS) data for atherosclerosis were obtained from UK Biobank (discovery cohort) and FinnGen (validation cohort) 5, 40. Details for the study population, description, ancestry, and genotyping have been described elsewhere (http://www.nealelab.is/uk-biobank; https://r10.finngen.fi/). For drug target Mendelian randomization (MR), cis-eQTLs with a linkage disequilibrium threshold of r2 < 0.1 were selected as instrumental variants. The TwoSampleMR (v0.6.0) package was used to identify the potential casual effect of KDM2A on atherosclerosis 41. The inverse-variance weighted (IVW) method with fixed- and random-effects model was calculated to obtain the MR effects estimates. The heterogeneity of the genetic instruments was assessed using the Q statistic. Additional analyses were performed, including simple mode, weighted mode, weighted median, and MR-Egger to account for horizontal pleiotropy. Colocalization analysis was performed using the "coloc" package 42. The colocalization analysis included five hypotheses and the posterior probability of H4 (PPH4) >0.5 was considered as supporting evidence for colocalization 43. The "LocusCompareR" package was used to visualize the colocalization region. In addition, the CVDKP platform was used to explore the potential association between KDM2A and cardiovascular and metabolic diseases (https://cvd.hugeamp.org/) 44.

### Virtual screening

The 3D structure of the human KDM2A protein (PDB ID: 4BBQ) was obtained from the Protein Data Bank (PDB, http://www.rcsb.org/pdb). Water and solvent molecules were removed, and hydrogen atoms as well as Gasteiger charges were added. Rotatable bonds were defined using AutoDock Tools. The grid box was configured with center coordinates (center_x: 5.04, center_y: 20.05, center_z: 65.16) and box size (size_x: 46, size_y: 46, size_z: 46) to encompass the whole protein. A Bioactive Compound Library (n=21321) (Library from medchemexpress company, https://www.medchemexpress.cn/screening/bioactive-compound-library-max.html) was screened using AutoDock Vina. Structural representations were generated using PyMOL software.

### Cell isolation and culture

Bone marrow-derived macrophages (BMDMs) were obtained as previously described 38. Briefly, after mice were anesthetized with ketamine (50 mg/kg^-1^) and xylazine (5 mg/kg^-1^), the skin was removed from abdomen and leg of the mice, the legs were separated at the hip joint, the muscles were dissected with a scalpel, and the tibia and femur were removed. The bone marrow of the tibia and femur was rinsed with PBS to collect macrophages. After lysing red blood cells with ammonium chloride-potassium lysing buffer, the cells were cultured in high-glucose DMEM medium containing 10% fetal bovine serum, 1% penicillin/streptomycin and macrophage-colony stimulating factor (M-CSF, 20 ng/mL, #576404, BioLegend). On days 4 and 6 of differentiation, the medium was supplemented with fresh medium containing 20 ng/mL M-CSF, adherent cells were considered as BMDMs. Human umbilical vein endothelial cells (HUVECs) were donated by the Department of Cardiothoracic Surgery, the First affiliated hospital of Chongqing Medical University. HUVECs were cultured in endothelial cell medium (Cat#1001, Sciencell, CA, USA), and three to five generations of HUVECs were used for experiments. The method of obtaining VSMCs is also described in previous studies 38. Briefly, the aorta was separated and the surrounding adipose tissue and adventitial tissue were removed. The mouse aortas were cut into small pieces, digested in DMEM containing type I collagenase for 12 hours, and free VSMCs cells were obtained, centrifuged at 3000 rpm for 5 minutes, and the precipitates were resuspended in DMEM medium containing 10% fetal bovine serum (FBS), and three to five generations of VSMCs were used for the experiment. Original RAW264.7 (mouse macrophage cell line) was purchased from Servicebio (Wuhan, China) and cultured in DMEM medium containing 10% FBS. Cells were treated with 20 μg/mL ox-LDL (Yiyuan Biotechnology, Guangzhou, China) for 24 hours for further *in vitro* experiments. AZD0530 (MedChemExpress, USA) was applied to BMDMs at 10μM.

### Plasmid transfection and RNA interference

BMDMs were transfected with specific small interfering RNA (siRNA) and plasmid. SiRNA of targeting Kdm2a (Si-KDM2A) and overexpression plasmid (OE-KDM2A) were designed through Suzhou Genepharma company in China. BMDMs were transiently transfected with siRNA oligonucleotides using transfection reagent (#AD600150, Zeta Life, USA) according to the manufacturer's protocol. And Invitrogen^®^ Lipofectamine^TM^ 3000 reagent was used to transfect plasmid according to the manufacturer's instructions. The siRNA KDM2A primer sequence is 5′-CCCGACGCAUGAACAAUAATT-3′. ox-LDL and AZD0530 were added after the transfected cells were cultured for 24 hours. Briefly, siRNA transfection or plasmid transfection was performed when the cell density reached 70%. SiRNAs or plasmids and transfection reagent were diluted with Opti-MEMTM medium (Cat# 31985070, Gibco, Thermo Fisher Scientific, USA) in tubes A and B, respectively. The liquids in tubes A and B were then mixed in equal proportions, and the mixture was incubated at room temperature for 15 minutes. The mixture was then added to the cell culture medium to initiate transfection and the medium was replaced with the fresh medium 6 hours later. Technical details can be found in our previous study 26.

### RNA-seq

BMDMs treated with ox-LDL for 24 hours under si-NC or si-KDM2A condition (5 vs 5) were collected for deep RNA-seq analysis (BGI Genomics, Shenzhen, China). The prepared mRNA libraries were sequenced on DNBseq (SE50) and filtered using SOAPnuke 45. Hisat v2 2.1.1 software was used to align the clean reads to the mm10 reference genome (https://ftp.ebi.ac.uk/pub/databases/gencode/Gencode_mouse/release_M23/gencode.vM23.annotation.gtf.gz). Then subread v2.0.6 software was then used to count the number of reads mapped to each gene (*featureCounts* function). The calculated genes with a total count greater than 1 were recognized as being expressed. Differential analysis was performed to explore the effect of KDM2A on macrophage function using the DESeq2 R package 46. Genes ordered by log2 (fold change) were used for GSEA analysis and the selected functional gene sets were visualized using the pheatmap R package.

### Reverse transcription-quantitative (RT-q) PCR

According to the manufacturer's instructions, total RNA was extracted using TRIZOL reagent, 1 ug of RNA was reverse transcribed using RT SuperMix, and real-time quantitative PCR was performed using SYBR Green PCR Master Mix (Q341-02, Vazyme) to detect the mRNA expression levels of relevant genes. β-ACTIN was used as an internal control for quantification, and relative RNA expression was analyzed using the 2-ΔΔCt method. Primers for qPCR are listed in Table 1.

### Western blotting

After the treatment, the cells were washed with PBS, and was added RIPA lysis buffer containing protease and phosphatase inhibitors. The cells were collected in a centrifuge tube, and centrifuged at 12000 rpm for 15 minutes, 4°C. After centrifugation, the protein supernatant was collected, and the total protein concentration was quantified using a BCA protein detection kit (Beyotime, China). 40 ug of total protein was used for Western blotting. The sample was mixed with 5× loading and heated it in a metal bath at 100°C for 10 minutes to denature the protein. Proteins were separated by SDS-PAGE and then transferred to a PVDF membrane. After blocking with 5% skim milk in TBST, the membrane was incubated with relevant antibodies at 4°C overnight. Membranes were incubated with HRP-conjugated secondary antibodies for 1 hour. The bands were then detected using a chemiluminescence imaging system and the grey scale values were quantified using Image J software.

### Animal study

8-week-old ApoE^-/-^ mice (C57 background, Cavens, Jiangsu, China) were housed in the Animal Center of Chongqing Medical University, maintained on a 12-hour light/dark cycle, and given free access to food and water. After one week of acclimatization, the mice were induced by feeding a Western-type high-fat, high-cholesterol (HFD) diet (D12109C, Research Diets) (AS group). To obtain early and late atherosclerotic lesions, mice in the AS group fed for 8 or 16 weeks were harvested. To validate the atherogenic function of KDM2A, 100μl adeno-associated virus serotype 9 (AAV9) of KDM2A (HBAAV2/9-F4/80-mir30-m-KDM2A-mcherry, M-KDM2A KD group) (1×10^11^ v.g) or negative control (HBAAV2/9-F4/80-mcherry, Control group) was injected through the tail vein at week 10 of high fat feeding. The AAV-associated virus was purchased from Han Hundsun Biological Technology. All animals were treated humanely, all animal procedures were in accordance with the protocol approved by the Animal Care and Use Committee of Chongqing Medical University (No. 2021-604), and all animal procedures complied with the relevant legal and ethical requirements.

After HFD feeding, all mice were euthanized. The aortic roots of the mice were then isolated and used to prepare the frozen sections. Aorta and carotid arteries were isolated and fixed in 4% paraformaldehyde. After dehydration, the tissues were embedded in paraffin wax and stained with hematoxylin and eosin (HE). Sections were visualized using a Leica DM4B microscope.

### Oil-red O staining

Aortic root sections from mice were stained with Beyotime (China), soaked in Oil-red O staining solution for 10 minutes, washed with washing solution, and then hematoxylin staining solution was added to stain the nucleus. The same procedure was applied to cells. RAW264.7 cells were cultured in 24-well plates, and after treatment, the medium was aspirated and washed with PBS. After fixation with 4% paraformaldehyde for 30 minutes, Oil-red O staining solution (Merck, 1320-06-5) was added for 10 minutes, and after washing with washing solution, the hematoxylin staining solution was added to stain the nucleus. Analysis was performed using a microscope and ImageJ software (described above).

### Immunofluorescence staining

Mouse aorta and human carotid atherosclerotic samples were harvested, fixed in 4% paraformaldehyde, embedded in paraffin, and sectioned at 5 um. Sections were dewaxed, rehydrated, and then boiled in citrate buffer for 5 minutes to extract antigens before being rinsed twice with PBS. To assess the expression of different proteins in the same tissue or cell section, tissue and cell sections were permeabilized with 0.1% Triton X-100 in PBS for 20 min, blocked with 5% goat serum for 1 h, and were then incubated with the respective target antibodies overnight at 4 °C. The sections were then treated with 4′,6-diamidino-2-phenylindole (DAPI) for 5 minutes, followed by incubation with multi-label fluorescence-conjugated secondary antibodies (AIFang biological, Hunan, China) for 1 hours at room temperature in the dark.

BMDMs are seeded in 24-well plates and at the end of the treatment, the medium was discarded and PBS was added for washing. After fixation with 4% paraformaldehyde for 30 minutes, 0.5% Triton X-100 was added for 20 minutes. Block with 10% goat serum for 30 minutes, discard blocking solution, add primary antibody and block overnight at 4°C. After washing the primary antibody with PBS, fluorescent secondary antibody was added and incubated at 37°C in the dark for 1 h. After incubation, the secondary antibody was washed with PBS, DAPI was incubated for 5 minutes and then observed under fluorescence microscopy (Leica Microsystems, Germany). ImageJ was used to qualify the number of positive cells and calculate the fluorescence intensity of the cells. The KDM2A antibody (Abcam, ab191387) and the inducible NO synthase (iNOS) antibody (Abcam, ab15323) were used for immunofluorescence.

### ChIP-seq and ChIP-qPCR

1×10^7^ ox-LDL treated RAW264.7 cells were collected for chromatin immunoprecipitation followed by sequencing (ChIP-seq) at GeneCreate Biological Engineering (Wuhan, China). Briefly, five nanograms of DNA were used as starting material for input and IP samples. The libraries were amplified for 13 cycles on the thermocycler. And the sequencing results were analyzed using BGI DNBSEQ-T7. Low quality reads were filtered out using fastp v0.20.0 software. After the quality control step, the clean reads were aligned to the mouse GRCm38 genome using Bowtie2 2.4.4 software (https://ftp.ncbi.nlm.nih.gov/genomes/all/GCA/000/001/635/GCA_000001635.8_GRCm38.p6/). Data quality was assessed using the deeptools v3.5.1 tool with the parameters "plotFingerprint --minMappingQuality 30 --skipZeros --numberOfSamples 50000". The MACS2 program (v2.2.7) was used to identify KDM2A binding peaks. Peak motif analysis was then conducted by homer software (v4.11.1) and peak annotation was performed using the chipseeker (v1.26.2) R package. Functional enrichment for peaks located near corresponding genes was further performed by clusterProfiler R package (v3.18.1). The significant target peaks of KDM2A were visualized through IGV 2.15.2 software.

RAW264.7 cells (4 × 10⁶ per immunoprecipitation) treated with ox-LDL were subjected to ChIP-qPCR using the SimpleChIP® Plus Kit (CST, 9005) according to the manufacturer's protocol. Immunoprecipitated DNA and DNA isolated from 2% input chromatin were amplified by quantitative PCR using SYBR Green PCR Master Mix (Q341-02, Vazyme) and locus-specific primers. Results were expressed as the percentage of input, calculated as the amount of DNA amplified from immunoprecipitated samples relative to the corresponding input DNA, using the same primers. The ChIP-qPCR primers used in this study are listed in Table 1.

### Antibodies

Primary antibodies against KDM2A (1:1000, 24311-1), NOX2 (1:5000, 19013-1), FYN (1:10000, 66606-1), BCL2 (1:1500, 26593-1), NRF2 (1:2000, 16396), SOD2 (1:10000, 66474-1) as well as secondary antibodies (goat anti-mouse, SA00001-1; goat anti-rabbit, SA00001-2) were purchased from Proteintech Group (Chicago, IL, USA); IL-1β (sc-12742, 1:2000) from Santa Cruz Biotechnology (Santa Cruz, CA, USA); TNF-α (1:500, AF7014), BAX (1:1000, AF0120), and acetyl-SOD2 (AC-SOD2) (1:1000, AF3751) were purchased from Affinity (USA). NQO1 (1:1000, 3187S), CD68 (1:1000, 97778S) and iNOS (1:1000, D6B6S) were purchased from Cell Signaling Technology (Beverly, MA, USA). Phospho-FYN (p-FYN) (1:1000, AF01116) was purchased from AiFang biological (Hunan, China). The KDM2A antibody used for immunofluorescence was purchased from Abcam (1:1000, ab191387).

### Statistical analysis

All statistics were calculated using the GraphPad Prism 9.0 software or R 3.6.3 or 4.2.1 software. *In vitro* experiments included at least 3 biological replicates. Spearman's rank correlation test was used for correlation analysis. Binary logistic regression analysis was used for clinical significance part (adjusted for demographic variables). Statistical significance between two groups was assessed using paired Student's t-tests for paired samples, and Mann-Whitney U-test or Wilcoxon signed-rank test for unpaired samples (where indicated). For more than two groups, statistical significance was assessed by one-way ANOVA by Tukey's multiple comparison test. A p-value of less than 0.05 was considered statistically significant.

## Results

### Identification of master regulons in atherosclerosis development

**Figure 1:**
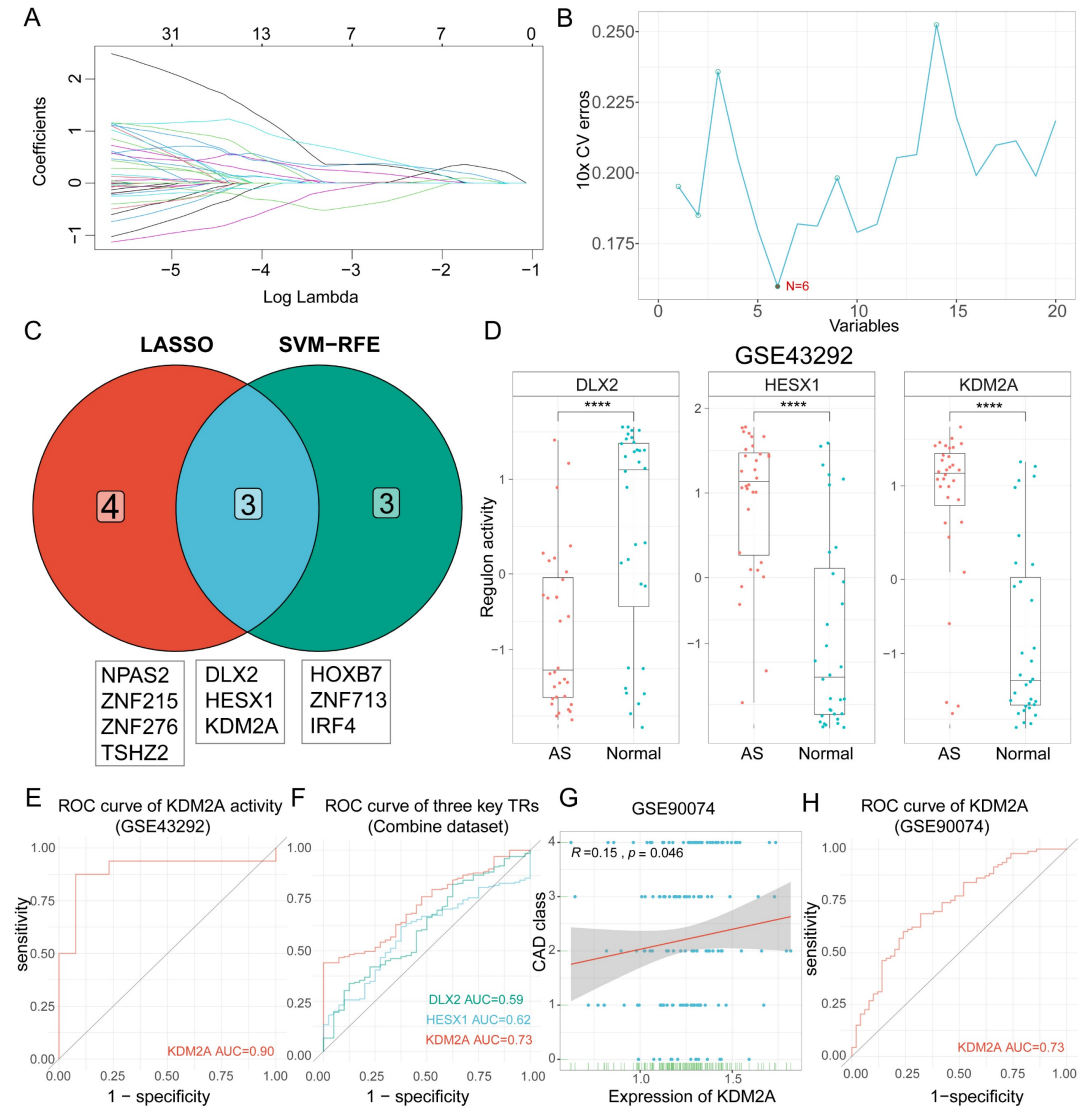
Identification of KDM2A as a key transcriptional regulator (TR) in the development of atherosclerosis. Screening of master regulons by (A) LASSO and (B) SVM-RFE machine learning algorithms. (C) Key regulons were identified as the intersection of these two algorithms. (D) Boxplots show the difference of the three TRs activities (DLX2, HESX1, and KDM2A) between macroscopically intact (n = 32) and atheroma plaque tissue (n=32) groups. (E) Receiving operating curve (ROC) shows the diagnostic efficacy of KDM2A regulon activity in GSE43292. (F) Validations of ROC curves (DLX2, HESX1, and KDM2A) in the combine dataset (n = 235 in total, AS = 195, normal = 40). (G) Linear regression plot of KDM2A expression with coronary artery disease (CAD) class in GSE90074 (n=143) (correlation coefficient = 0.15, P value = 0.046). (H) Receiving operating curve (ROC) shows the diagnostic efficacy of KDM2A regulon activity in GSE90074 (n = 143 in total, non-obstructive = 50 and obstructive = 93). Correlation analysis was examined by Spearman's test. Expressions were presented in boxplots together with the group mean ± SE. For two groups, data were compared by paired t-test test: ****P < 0.0001.

Atherosclerosis is characterized as a lipid-driven inflammatory damage of the arterial intima, and the complexity of the functional response is determined by triggered TRs. A total of 1468 regulatory networks with regulon activities were constructed. After differential analysis, 404 regulons were dysregulated in plaque compared to the normal group (Supplementary table 2), of which 136 regulons were upregulated and 268 regulons were downregulated (Supplementary figure 1A). The top 10 up- and downregulated regulons are shown in Supplementary figure 1B. Accordingly, two different two different techniques for feature selection (LASSO and SVM-RFE) were applied to identify the potent regulons of atherosclerosis development (Figure 1A-B). Seven (NPAS2, ZNF215, ZNF276, TSHZ2, DLX2, HESX1, and KDM2A) and six (HOXB7, ZNF713, IRF4, DLX2, HESX1, and KDM2A) regulons were identified by LASSO and SVM-RFE, respectively. After intersection analysis, three regulons (DLX2, HESX1, and KDM2A) were identified as master TRs in atherosclerosis (Figure 1C). The regulatory networks were visualized in Supplementary figure 1C, in which 72 target genes of KDM2A, 19 target genes of HESX1, and 6 target genes of DLX2 were pruned by RTN analysis (Supplementary table 3). The box plot showed that DLX2 was inactivated in the plaque (AS group), whereas HESX1 and KDM2A were activated in the AS group based on GSE43292 (Figure 1D). Then, differences in the activities of the three regulons were validated in the combine dataset (Supplementary figure 1D). Only KDM2A was still activated in AS group with an excellent distinguish ability in GSE43292 (area under curve, AUC = 0.90) and the combine dataset (AUC=0.73, Figure 1E-F).

The transcriptional changes of the three TRs were also examined during atherosclerosis progression (GSE28829, including early atherosclerotic and advanced atherosclerotic plaque samples, n = 13 vs. n =16, respectively). KDM2A was still elevated in the advanced atherosclerotic plaque group (Supplementary figure 1E). To further validate the diagnostic efficacy of KDM2A, correlation and regression analyses were performed for the association of KDM2A levels from PBMCs with CAD class. We observed that KDM2A level in PBMCs is strongly correlated with CAD class (coefficient = 0.15,* P* = 0.046) and a good predictor for CAD classification (non-obstructive vs. obstructive, AUC = 0.73) (Figure 1G-H), instead of DLX2 and HESX1 (Supplementary figure 1F-G). The boxplot also showed the significant increase of KDM2A in obstructive CAD patients (Supplementary figure 1H). In the adjusted model, KDM2A was consistently associated with the risk of obstructive CAD (odds ratio = 6.00 [95% confidence interval, 1.03-34.91], *P* = 0.046) (Supplementary figure 1I). Therefore, we believe that KDM2A expression, especially in major immune cells (T cells or macrophages), is significantly associated with atherosclerosis, suggesting that KDM2A expression is potentially linked to immune response. Furthermore, we performed functional enrichment analysis of the target genes of KDM2A using the Metascape, and the top terms indicated the potential association of KDM2A with inflammation and immune cell response (Supplementary figure 1J).

### Involvement of KDM2A in macrophage function

**Figure 2:**
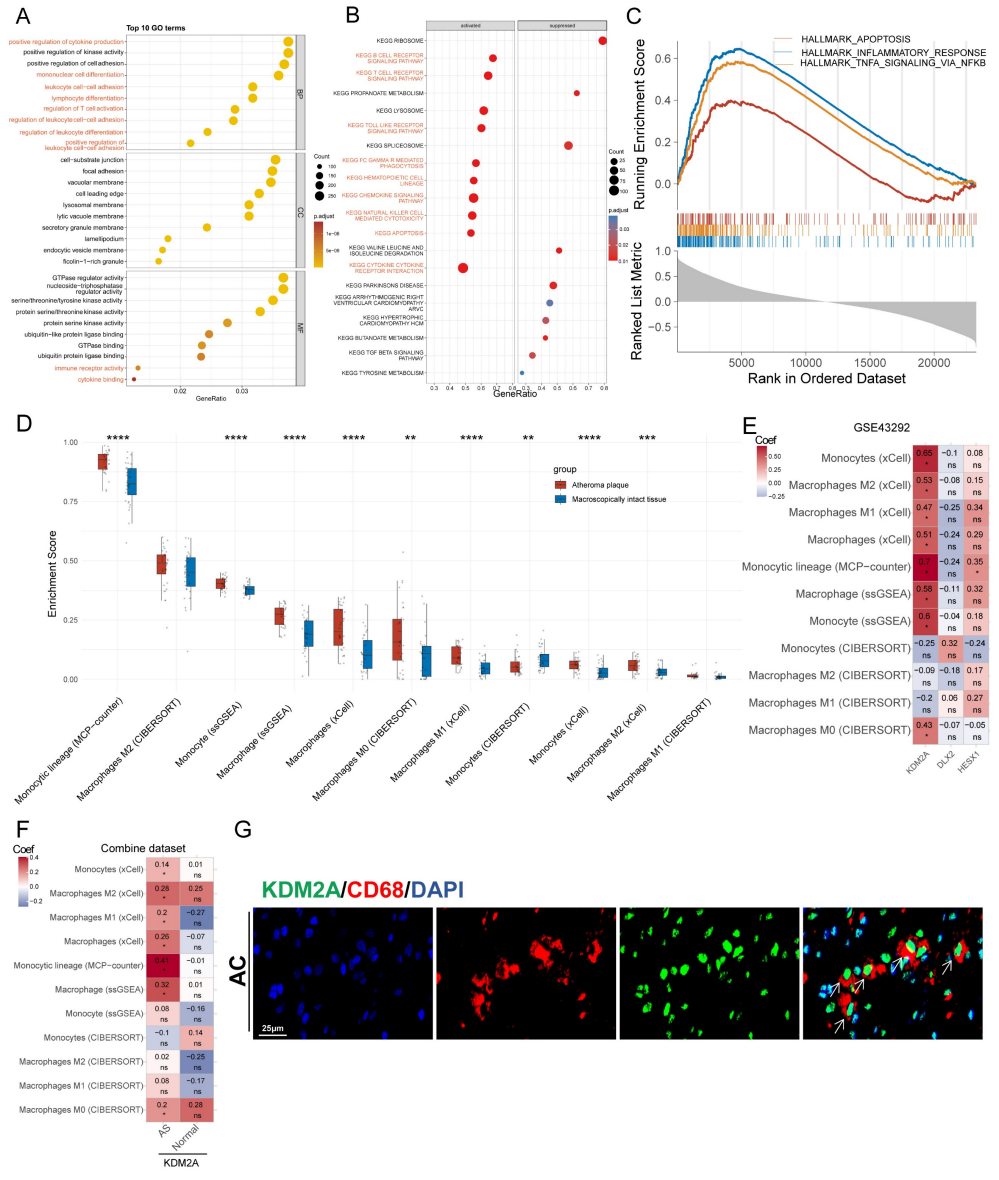
KDM2A is involved in macrophage activation. Functional delineation of KDM2A by (A) gene ontology (GO), (B) Kyoto Encyclopedia of Genes and Genomes (KEGG), and (C) gene set enrichment analysis (GSEA). (D) The differences in the proportions of macrophage subtypes inferred by four algorithms (CIBERSORT, ssGSEA, xCell, and MCP-counter) in GSE43292 (n = 32 per group). (E) Correlation heatmap of three TRs (HESX1, DLX2, and KDM2A) with abundances of 11 macrophage subtypes in plaque samples (n = 32) of GSE43292. (F) Correlation heatmap of KDM2A with proportions of 11 macrophage subtypes in plaque (n = 195) and normal (n = 40) samples in the combine dataset. (G) Representative immunofluorescence staining images for KDM2A and CD68 in carotid arteries from a patient with atherosclerosis. Expressions are presented as box plots showing the group mean ± SE. Data for two groups were compared by paired-t test: **P < 0.01, ***P < 0.001, ****P < 0.0001.

To precisely define the potential functions of KDM2A in atherosclerosis, GO, KEGG, and HALLMARK GSEA analyses of genes correlated with the regulon activity of KDM2A were performed. In the biological process subset, 8 of the top 10 terms were associated with immune cell activation (labelled in red in Figure 2A, Supplementary table 4). KEGG analysis also indicated that KDM2A was involved in inflammatory pathways, such as B- and T-cell receptor signaling, chemokine signaling, cytokine-cytokine receptor interactions, and FC gamma R-mediated phagocytosis, etc. (Figure 2B, Supplementary table 5). HALLMARK terms, including INFLAMMATORY_RESPONSE (most prominent, NES = 3.12), TNFA_SIGNALING_VIA_NFKB (NES = 2.82), and APOPTOSIS (NES = 1.86), were activated, indicating that KDM2A is significantly involved in the inflammatory response of immune cells (Figure 2C and Supplementary table 6).

We therefore investigated the relationship between the potential regulons and the abundance of immune cells in plaque samples. KDM2A activity was positively associated with 24 out of 28 immune cell proportions (Supplementary figure 2A) using the ssGSEA algorithm. Accounting for the fact that macrophages and T cells constitute the main components of the innate and adaptive immune responses and play crucial roles in the development of atherosclerosis, we focused on different macrophage and T cell subtypes 47-50. First, we found that most macrophage subsets from four different algorithms (except for M2 subtypes) were more abundant in the AS group compared with normal artery (Figure 2D), and the differences were consistent in the combine dataset (Supplementary figure 2B). Furthermore, KDM2A activity was strongly associated with different macrophage subsets by different algorithms (except for CIBERSORT) (Figure 2E and Supplementary figure 2C). We also found that the significantly positive associations were not maintained in normal arteries (Figure 2F), indicating the potential correlation of KDM2A activation and macrophage inflammatory response. As for T cells, we also found that most T cell subtypes were upregulated in plaque (Supplementary figure 2D and 2E). However, the associations of KDM2A activity with T cell subtypes were not consistent across different algorithms and datasets (Supplementary figure 2F and 2G). For example, although different T cell subtypes (such as regulatory, Th1, gamma delta, and CD8+ T cell) are positively correlated with KDM2A activity through ssGSEA analysis, the relationships could not be validated by other algorithms (MCP-Counter, XCell, and CIBERSORT) (Supplementary figure 2G). Therefore, the regulatory role of KDM2A in T cell isn't convincing. Furthermore, in a representative human plaque lesion, KDM2A+ cells were co-expressed with CD68 (Figure 2G), confirming our analysis.

### scRNA-seq data reveals the role of KDM2A in macrophage phenotypical alterations

**Figure 3:**
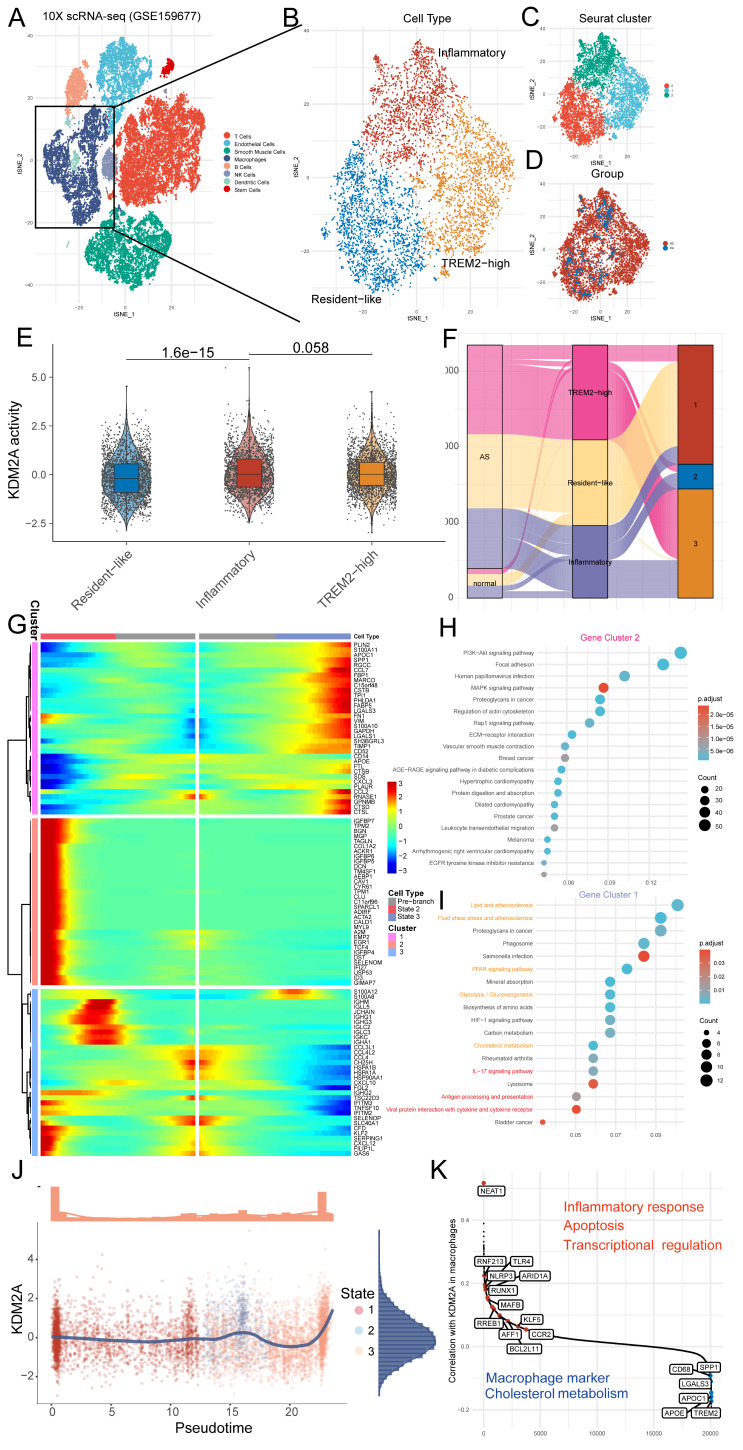
KDM2A participates in the functional state alteration of macrophages. tSNE clustering plots showing (A) 8 cell populations of GSE159677 (a total of 35,021 individual cells), (B) 3 macrophage subtypes, (C) 3 color-coded cell clusters, and (D) different groups (of 6533 individual cells). (E) Violin/box plot of KDM2A activities across different macrophage subtypes. (F) Sankey diagram linking cell types and different Monocle states between atherosclerotic and normal groups. (G) Heatmap showing the expression dynamics of 3 gene clusters with the first branch point determined by BEAM analysis. Kyoto Encyclopedia of Genes and Genomes (KEGG) analyses of gene clusters (H) 2 and (I) 1 belongs to State 2 and 3, respectively. (J) Pseudotime kinetics of KDM2A activity with pseudotime progression (colored by different State). (K) Displayed genes ordered by correlation coefficient with KDM2A activity. Correlation analysis was examined by Spearman's test; for two groups, data were compared by Wilcoxon signed-rank test. Expressions are presented as box plots showing the group mean ± SE.

Using the GTEx database, we validated the expression of KDM2A in different organs/tissues. We found that KDM2A is expressed at the highest levels in blood vessels, with the exception of the lungs (Supplementary figure 3A). We also investigated the potential function of macrophage KDM2A based on scRNA-seq. A total of 35,021 cells were clustered into 19 cell populations and 5 main cell types were identified based on the automatic and manual annotation (Figure 3A and Supplementary figure 3B-C) as we previously described 38. Among the main cell types (>3%) in the plaque, KDM2A expression was higher in macrophages than in T cells (Supplementary figure 3D). To explore the specific role of KDM2A in macrophages, we extracted cells annotated as macrophages for sub-clustering. Cluster 0, which showed upregulation of *F13A1* as well as the anti-inflammatory markers *FOLR2*, was identified as a resident subset (Figure 3B-D, Supplementary figure 4A) 51. Cluster 1 was recognized as TREM2-high (high expression in *TREM2* and *LGALS3*) (Figure 3B-D, Supplementary figure 4A) 52, 53. In addition, cluster 2 showed a predominant upregulation of inflammation-associated genes (*IL1B*, *S100A8*, *CXCL2* etc.), suggesting as an inflammatory phenotype (Figure 3B-D, Supplementary figure 4A) 54. The significant increase in the TREM2-high proportion and relative decrease in the resident-like subtype indicated a foaming process in the AC group (Supplementary figure 4B). DoRothEA, an efficient tool for inferring TR network activities in single-cell resolution, was implemented in our study 55. Compared to resident-like subtype, KDM2A activity was significantly upregulated in inflammatory and TREM2-high subtypes, indicating KDM2A activation in macrophage phenotypic alterations (Figure 3E). Although KDM2A activity was relatively higher in inflammatory macrophages (inflammatory vs. TREM2-high, *P* = 0.058), the insignificance shown suggests that KDM2A may play a role in both inflammatory and TREM2-high subtypes. Therefore, the Monocle analysis was utilized to elucidate the underlying biological processes of KDM2A across different functional states.

Three different functional states of macrophages were discovered by trajectory inference (Supplementary figure 4C-F). Obviously, resident-like macrophages were predominantly included in state 1 (98.17%), while most inflammatory and TREM2-high cells constituted the majority of state 2 and 3 (75.54% and 65.41%, respectively) (Figure 3F and Supplementary table 7). Furthermore, the increased abundances of state 3 (19.41% to 46.68%) and decreased proportions of state 2 (34.04% to 8.19%) in AC samples compared to PA samples indicated the state alterations of macrophages in atherosclerosis (Supplementary figure 4G and Supplementary table 8). Accordingly, we performed BEAM analysis to identify the genes involved in the differentiation of the trajectory of the first point. The genes were assigned into 3 gene clusters with different branch-dependent expression pattern (Figure 3G and Supplementary table 9). Gene cluster 2, upregulated in state 2, was associated with PI3K-Akt, focal adhesion, regulation of actin cytoskeleton, ECM-receptor interaction signaling (Figure 3H). Gene cluster 1, highly expressed in state 3, was associated with lipid-related metabolism (including lipid and atherosclerosis, fluid shear stress and atherosclerosis, cholesterol metabolism, glycolysis, and PPAR signaling) and inflammatory (including antigen processed and presentation, viral protein interactions with cytokine and cytokine receptor, and IL-17 signaling) pathways (Figure 3I). Furthermore, regression analysis of KDM2A activity with pseudotime revealed that KDM2A shifted sharply at the late stage of the macrophage trajectory (predominantly comprising state 3 cells) (Figure 3 J). In addition, we also observed the drastically upregulated levels of KDM2A activity in state 3 (Supplementary figure 4H). These data likely demonstrated the potential association of KDM2A with macrophage lipid metabolism and functional activation. Correlation analysis of KDM2A activity also showed positive associations with inflammatory response, apoptosis, and transcriptional regulation markers (including *NEAT1*, *CCR2*, *NLRP3*, *TLR4*, *BCL2L11*, *MAFB, RNF213*, *ARID1A*, *RUNX1*, *RREB1*, and *AFF1*) but negative associations with macrophage marker and cholesterol metabolism related genes (*CD68*, *SPP1*, *LGALS3*, *APOC1*, *APOE*, and *TREM2*) (Figure 3K and Supplementary table 10) 56-61.

We also observed high expression of S100A8/9 in inflammatory macrophages, typically considered neutrophil markers. However, examination of other neutrophil markers (FUT4, CXCR2, GNL2, S100P, CAMP, MPO) revealed negligible expression in macrophage subtypes (Supplementary Figure 4I) 62. In addition, immunofluorescence staining of mouse plaques revealed neutrophil infiltration (Ly6G+ cells); however, these cells did not co-express KDM2A, indicating that KDM2A is primarily expressed in macrophages (Supplementary figure 4J).

Additionally, GSE260657, generated by SmartSeq2 with a total of 7,686 cells clustered into 12 populations and defined as 6 main cell types after quality control, was used to validate the potential function of KDM2A (Supplementary Figure 5A-D and Supplementary Table 11). Among 6 main cell types (>3%) in plaque, we found that KDM2A expression was significantly upregulated in myeloid cells (Supplementary Figure 5E). Further sub-clustering analysis of the myeloid cells also identified three main macrophage subtypes: Resident, inflammatory, and TREM2-high, in the Smart-seq2-based scRNA-seq data (Supplementary Figure 5F-J and Supplementary Table 12). KDM2A expression was also significantly upregulated in the inflammatory subtype (Supplementary Figure 5K). Based on TRs activity inferred by the Dorothea algorithm, KDM2A activity was positively correlated with inflammatory response and negatively correlated with lipid metabolism and classical macrophage markers (Supplementary Figure 5K and Supplementary Table 13).

### KDM2A is the key TR of macrophage inflammatory response

**Figure 4:**
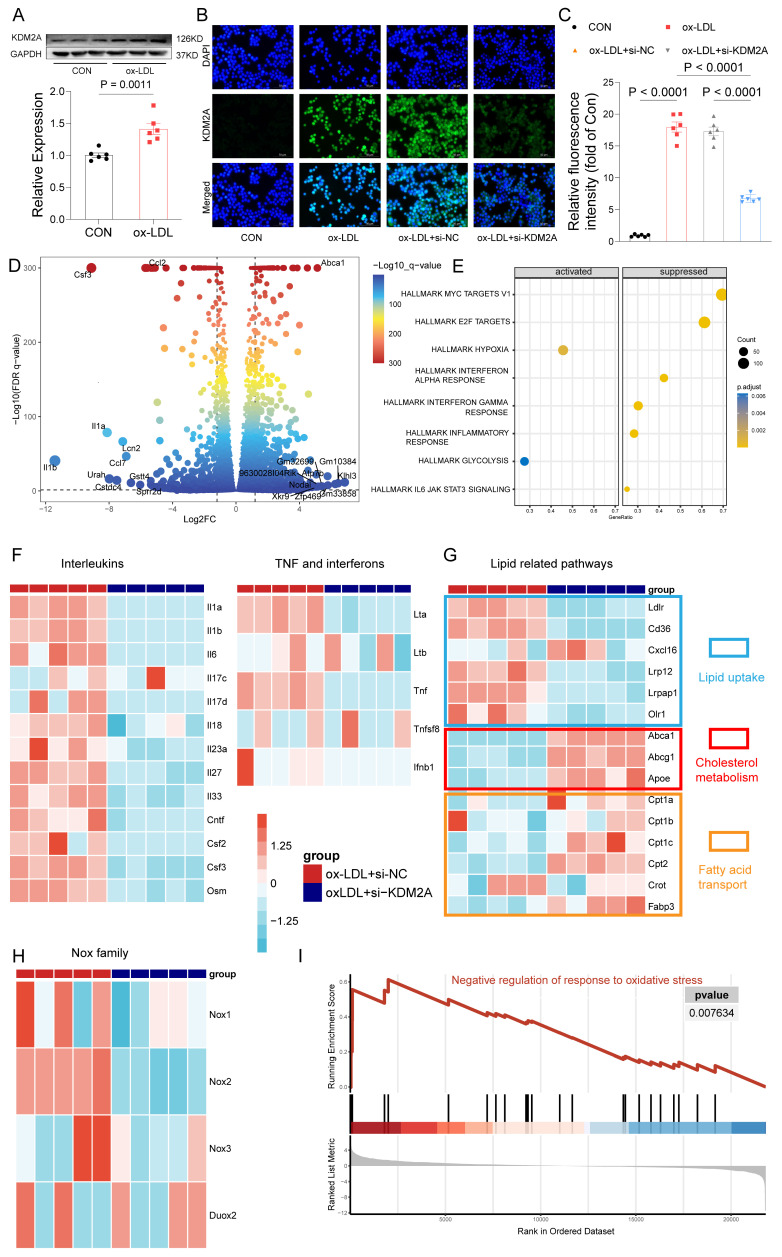
KDM2A regulates the inflammatory response of macrophages. (A) Western blot assay demonstrates significant expression of KDM2A in bone marrow-derived macrophages (BMDMs) cultured with 20 ug/ml ox-LDL for 24 h. Data were shown as bar plot compared by Mann-Whitney U-test. (B) Immunofluorescence staining reveals alterations in KDM2A expression under siRNA Kdm2a (Si-KDM2A) treatment and quantified by (C) bar plot. Data were compared by one-way ANOVA test. (D) Differentially expressed genes identified between si-KDM2A treatment (n=5) and si-NC (n=5) BMDMs under ox-LDL treatment, represented by color and size dots in a volcano plot. (E) HALLMARK gene set enrichment analysis (GSEA) terms of differentially expressed genes after KDM2A knockdown. Heatmap shows the normalized read counts (log transformed) of genes belonging to (F) cytokines, (G) lipid-related pathways, and (H) Nox family. (I) GSEA plot showing upregulation of *negative regulation of response to oxidative stress* in si-KDM2A group. Expressions are presented as bar plots showing the group mean ± SEM, with n=6 per group.

Given the demonstrated association of KDM2A with macrophage phenotypic alterations in plaque, we further validated the functions of KDM2A by *in vitro* experiments. Initially, although our focus was on the role of KDM2A in macrophages, we also examined the KDM2A levels in non-immune cells under ox-LDL treatment. We found that KDM2A expression in HUVECs remained unchanged across different concentrations of ox-LDL (Supplementary figure 6A) and different time points (Supplementary figure 6B). Similarly, the expression levels of KDM2A in VSMCs were also unchanged under different concentrations of ox-LDL at 24 hours (Supplementary figure 6C). Next, we examined the levels of KDM2A in BMDMs under different concentrations of ox-LDL (Supplementary figure 6D), and Kdm2a protein levels exhibited a significant increase at 20 ug/ml of ox-LDL. Meanwhile, the protein levels of Kdm2a were continuously upregulated at 12 h and further time points, as well as transcriptional levels (Supplementary figure 6E-F), so an ox-LDL concentration of 20 ug/ml and a treatment timepoint of 24 h were applied in further experiments (Figure 4A). Mus-1969 was selected for subsequent analyses due to its noteworthy efficacy in silencing Kdm2a expression in macrophages (hereafter referred to as si-KDM2A) (Supplementary figure 6G). Using immunofluorescence, we observed an upregulation of KDM2A in the ox-LDL group, which was downregulated after si-KDM2A treatment. Additionally, we found that Kdm2a was primarily localized in the nucleus (Figure 4B-C). To gain insight into the mechanism by which Kdm2a regulates macrophage inflammatory response, we performed RNA-seq analysis on BMDMs treated with either si-NC or si-KDM2A under ox-LDL stimulation. The si-KDM2A group was characterized by 1937 overexpressed and 1079 downregulated genes compared to si-NC treatment (Figure 4D and Supplementary table 14). We observed that cytokines and chemokines, such as Il1b, Il1a, and Ccl2, were most significantly suppressed, whereas energy and cholesterol metabolism markers, including Atp7b and Abca1, were strongly activated after si-KDM2A treatment. GSEA analysis of HALLMARK terms also indicated that inflammation-related pathways were repressed and glycolysis was activated (Figure 4E and Supplementary table 15). We further analyzed those genes relevant to cytokines (including interleukins, TNF family, and interferon) and lipid metabolism. Apart from Il1a and Il1b, other cytokines were also significantly downregulated in si-KDM2A group (Figure 4F). Interestingly, genes correlated with lipid uptake were downregulated whereas genes involved in cholesterol metabolism and fatty acid transport were upregulated after si-KDM2A treatment (Figure 4G), suggesting that activation of KDM2A would lead to lipid accumulation. Previous studies have demonstrated that oxidative stress can induce macrophage inflammation and subsequently destabilize plaque 63-65. Accordingly, we analyzed the genes of the nicotinamide adenine dinucleotide phosphate (NADPH) oxidase complex (Nox1-3 and Duox2), which are the major donors of reactive oxygen species (ROS). These genes were downregulated in si-KDM2A group (Figure 4H). The oxidative stress-related GO terms also indicated the activation of negative regulation of the response to oxidative stress (Figure 4I and Supplementary table 16). Furthermore, the potential apoptosis and transcriptional regulation target genes of KDM2A through RTN and scRNA-seq were confirmed by RT-qPCR (Supplementary figure 6H).

**Figure 5:**
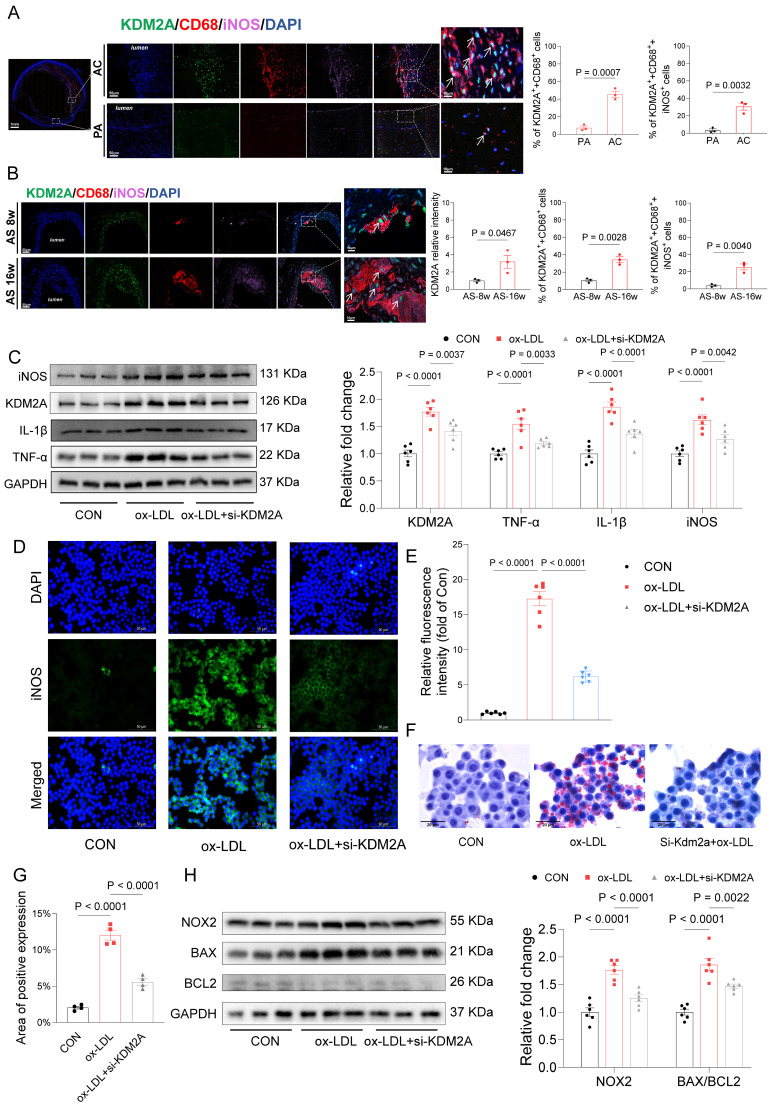
*In vitro* experiments confirm the pro-inflammatory role of KDM2A. (A) Quantification of the number of cells expressing KDM2A co-expressed with CD68 and iNOS within the human carotid samples. (B) Quantification of the number of cells expressing KDM2A co-expressed with CD68 and iNOS within the mouse plaque lesions. Data were shown as bar plots together with group mean ± SE and compared using the Mann-Whitney U-test (n=3 per group). (C) Western blot assay showed significantly increased expression of inflammatory markers (TNF-α, IL-1β, and iNOS) in ox-LDL treated bone marrow-derived macrophages (BMDMs), which was rescued by si-KDM2A. (D) Immunofluorescence staining showed decreased iNOS expression under Si-KDM2A and quantified by (E) bar plot (n=3 per group). (F) Oil-red O staining determined the lipid accumulation of RAW264.7 cells with si-KDM2A treatment, and quantified by (G) bar plot (n=3 per group). (H) Western blot assay showed that the increased oxidative marker (NOX2) and apoptotic markers (BAX and BCL2) were alleviated by si-KDM2A. Expressions are presented as bar plots showing the group mean ± SEM, with n=6 per group unless otherwise specified. Data for more than two groups were compared by one-way ANOVA test.

Based on carotid tissue samples from patients who underwent carotid endarterectomy, we compared the distribution of KDM2A and their co-localization with CD68 and iNOS in the PA and AC regions. Our findings indicate a significant increase in the nuclear accumulation of KDM2A in macrophages (CD68+ cells) and, notably, in inflammatory macrophages (CD68+iNOS+ cells) within the AC regions (Figure 5A). In the plaques of aortic arteries from ApoE^-/-^ mice fed an HFD diet, increased nuclear accumulation of KDM2A in macrophages was observed during plaque progression (Figure 5B). Furthermore, the proportion of KDM2A+ macrophage co-expressing with iNOS was also increased in the AS-16w group (Figure 5B). Western blot assays confirmed that the increased levels of inflammatory markers (TNF-α, IL-1β, iNOS) after ox-LDL treatment could be rescued by Kdm2a knockdown (Figure 5C). Consistently, in vitro immunofluorescence confirmed the pro-inflammatory function of KDM2A (Figure 5D-E). Considering the effect of KDM2A on lipid metabolism, Oil-Red O staining revealed that lipid accumulation in macrophages could be alleviated by KDM2A knockdown (Figure 5 F-G). Furthermore, NOX2 was also positively regulated by KDM2A, as well as apoptotic markers (BAX and BCL2) (Figure 5H), indicating that KDM2A also regulates redox homeostasis and apoptosis.

### KDM2A transcriptionally regulates FYN

**Figure 6:**
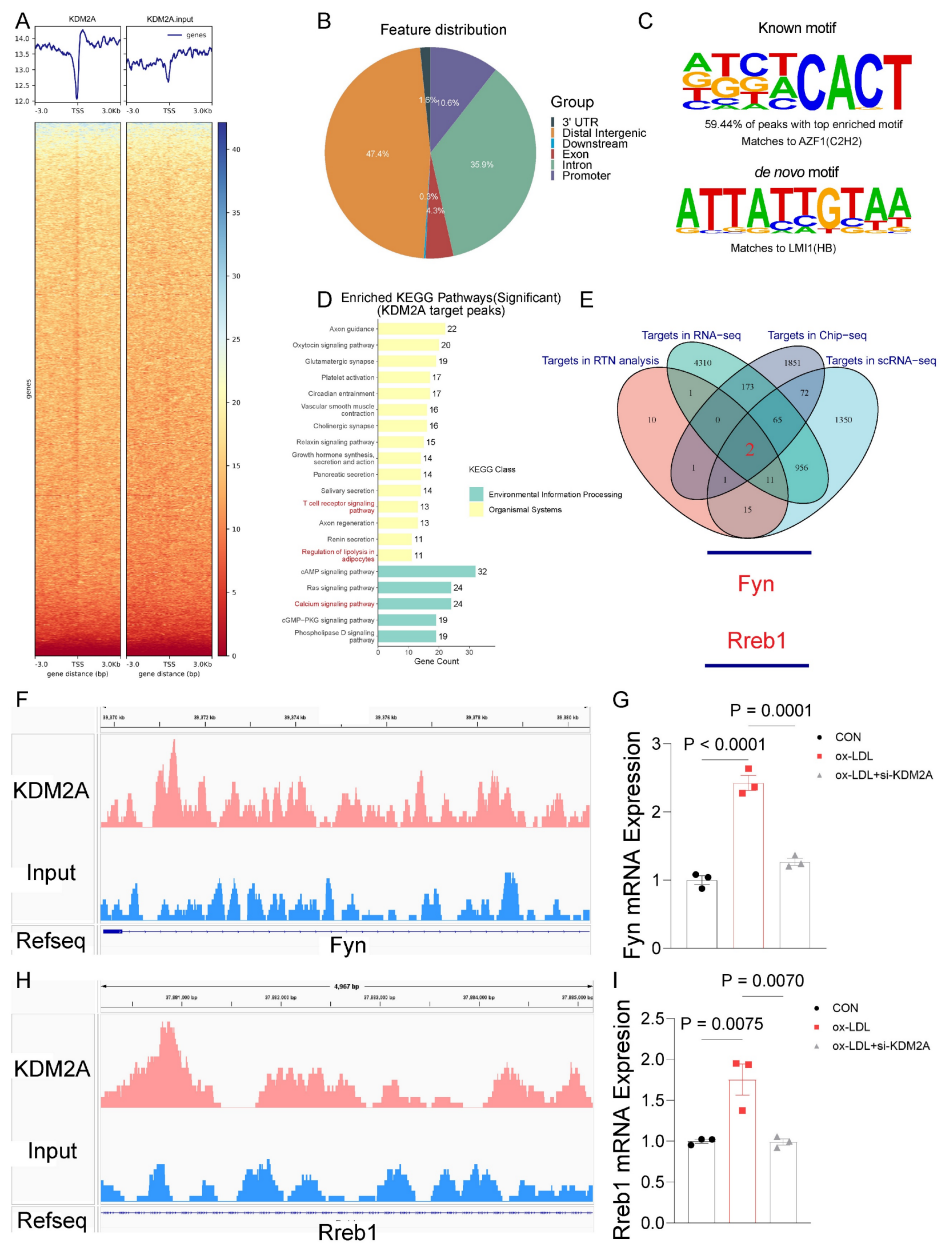
FYN and RREB1 are direct target genes of KDM2A in macrophages. (A) Average ChIP-seq signal distribution near the transcriptional start site (TSS). (B) Genome-wide distribution of KDM2A ChIP-seq peaks in RAW264.7 cells. (C) Motif enrichment analysis of KDM22A co-bound peaks. (D) KEGG analysis of genes regulated by KDM2A. (E) A Venn diagram showing the target genes involved in inflammatory response regulated by KDM2A, identified as the intersection of RTN, scRNA-seq, RNA-seq, and ChIP-seq analysis. Genome browser views of 4 kb genomic loci of the (F) Fyn and (H) Rreb1 showing ChIP-seq tracks with called peaks (red bars). The increased RNA expression of KDM2A target genes (G) Fyn and (I) Rreb1 could be reversed by si-KDM2A. Expressions are shown as group mean ± SEM in bar plots. Data were compared by one-way ANOVA test, with n=3 per group.

Having identified the pro-inflammatory role of KDM2A in macrophages, we performed further ChIP-seq experiments to identify the direct target genes of KDM2A under ox-LDL treatment (Figure 6A and Supplementary table 17). Most of the ChIP peaks were located at distal intergenic (47.4%), intron (35.9%), promoter (10.6%), and exon (4.3%) (Figure 6B and Supplementary table 18). And the motif of KDM2A targeted peaks was identified (Figure 6C). After enrichment analysis, peaks were enriched in calcium homeostasis based on GO terms (Supplementary figure 6 7A and Supplementary table 19), and genes were enriched in T cell receptor, chemokine signaling, leukocyte trans-endothelial migration, and regulation of lipolysis in adipocytes pathways based on KEGG terms (Figure 6D and Supplementary table 20). Intersection of RTN, RNA-seq, scRNA-seq, and ChIP-seq analyses suggested that Fyn and Rreb1 were the direct positively regulated genes by KDM2A (Figure 6E). The significantly different peaks were located at the promoter of Fyn and the intron of Rreb1 (Figure 6F and 6H), respectively. The positive regulatory relationships were also confirmed by RT-qPCR (Figure 6G and 6I). Furthermore, the positive associations of KDM2A with FYN and RREB1 were validated using plaque samples (Supplementary figure 7B-C). We then examined the correlations of KDM2A with FYN and RREB1 in 31 different organs-based GTEx. The associations (KDM2A-FYN and KDM2A-RREB1) was almost the most significant in blood vessels (Supplementary figure 7D-E).

**Figure 7:**
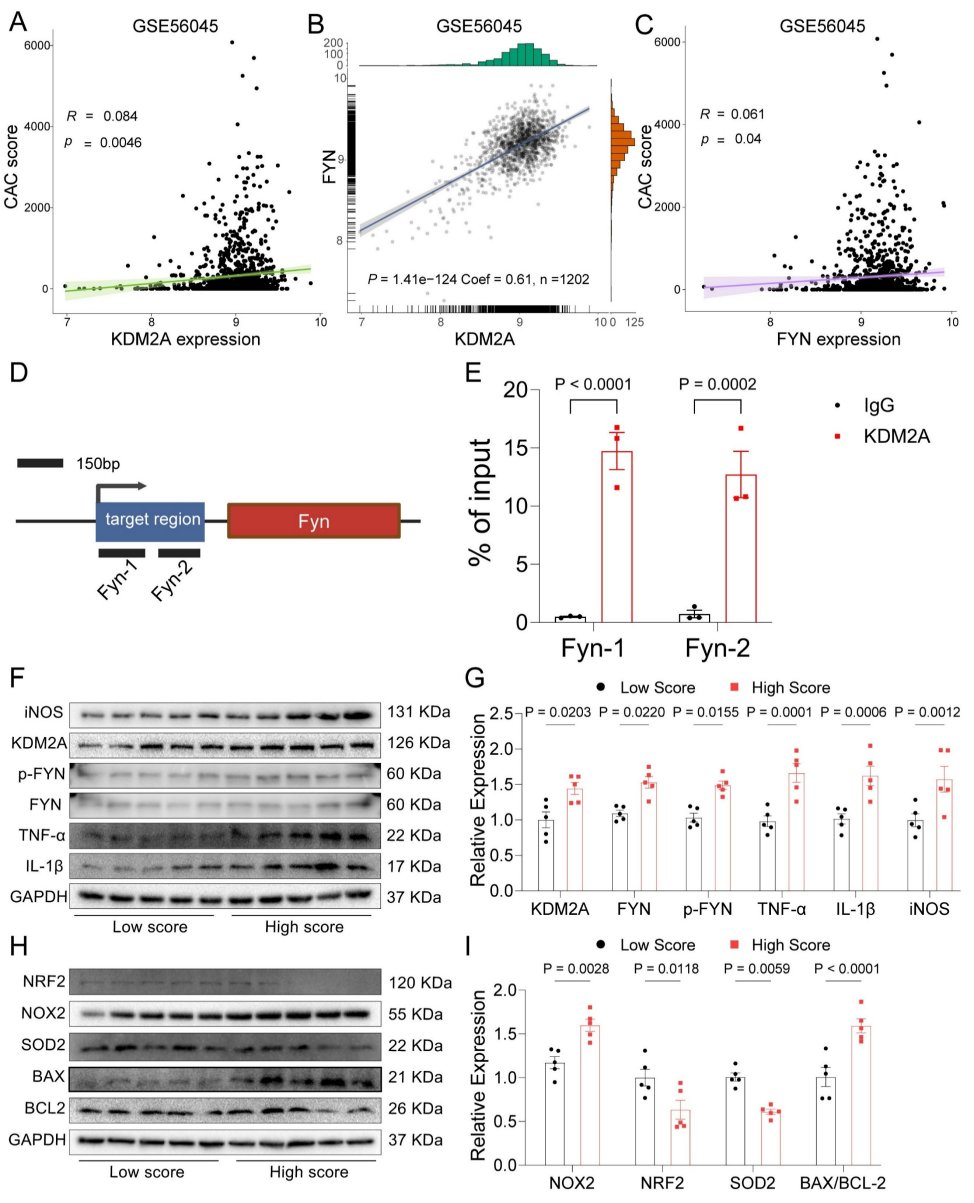
KDM2A transcriptionally regulate FYN. The association of KDM2A expression with (A) CAC score and (B) FYN expression in GSE56045. (C) The association of FYN expression with CAC score. (D) Schematic illustrated the design for *Fyn* primers. (E) ChIP-qPCR showed the direct binding of KDM2A with the promoter of *Fyn* (n=3 per group). (F-I) Peripheral blood mononuclear cells from stable CAD patients undergoing percutaneous coronary intervention were used to validate the dysregulation of KDM2A, p-FYN/FYN ratios, and associated pathways based on the Gensini score. The expression levels of inflammatory markers (TNF-α, IL-1β, iNOS), oxidative stress markers (NRF2, NOX2, SOD2), and apoptosis-related proteins (BAX and BCL2) were assessed by immunoblotting (n = 5 per group). Data are presented as bar plots showing the group mean ± SEM and compared by Mann-Whitney U-test. Correlation analysis was examined by Spearman's test.

Given the important role of KDM2A in macrophage inflammatory response during atherosclerosis development, we investigated the association of its expression in peripheral monocytes/macrophages with coronary/carotid atherosclerotic severity. The expression levels of KDM2A showed a strongly positive association with CAC score (*P* = 0.0046, R = 0.084) and carotid plaque severity (*P* = 0.011, R = 0.076) (Figure 7A and Supplementary figure 8A). The regulatory relationship between KDM2A and FYN was also confirmed in GSE56045 (Figure 7B). Furthermore, the FYN expression was also positively associated with CAC score (*P* = 0.04, R = 0.061) and carotid plaque severity (*P* = 0.12, R = 0.047) (Figure 7C and Supplementary figure 8B). Although the positive correlation between KDM2A and RREB1 expression was also observed (Supplementary figure 8C), there are no significant associations between RREB1 and CAC score (*P* = 0.96, R = 0.00014) and carotid plaque severity (*P* = 0.45, R = -0.023) (Supplementary figure 8D-E). Therefore, we further focus on the KDM2A-FYN axis in the macrophage inflammatory response. Previous studies had shown that H3K36me2 recruits DNMT3A and induces the DNA methylation of its target genes, which shapes the transcriptional processes in disease development 66, 67. Accordingly, we analyzed the potential effect of KDM2A on FYN methylation. We found almost no association of KDM2A with CpG sites in FYN, especially in the promoter region (Supplementary figure 8F), suggesting that the regulatory relationship of KDM2A is independent of DNA methylation. ChIP-qPCR experiments confirmed the direct binding of KDM2A to the *Fyn* promoter (Chr10:39371081-39371369) (Figure 7D-E and Supplementary Figure 8G-H). To complement transcriptional analysis, we performed WB analysis to assess KDM2A dysregulation at the protein level in patients with stable CAD who undergoing percutaneous coronary intervention. Notably, patients with a high Gensini score demonstrated pronounced systemic inflammation (Supplementary Figure 8I-K), elevated KDM2A expression, and increased p-FYN, FYN levels (Figure 7F-I). Additionally, they showed heightened markers of inflammation and oxidative stress (Figure 7F-I and Supplementary Table 21).

### KDM2A-FYN axis promotes macrophage inflammatory response

**Figure 8:**
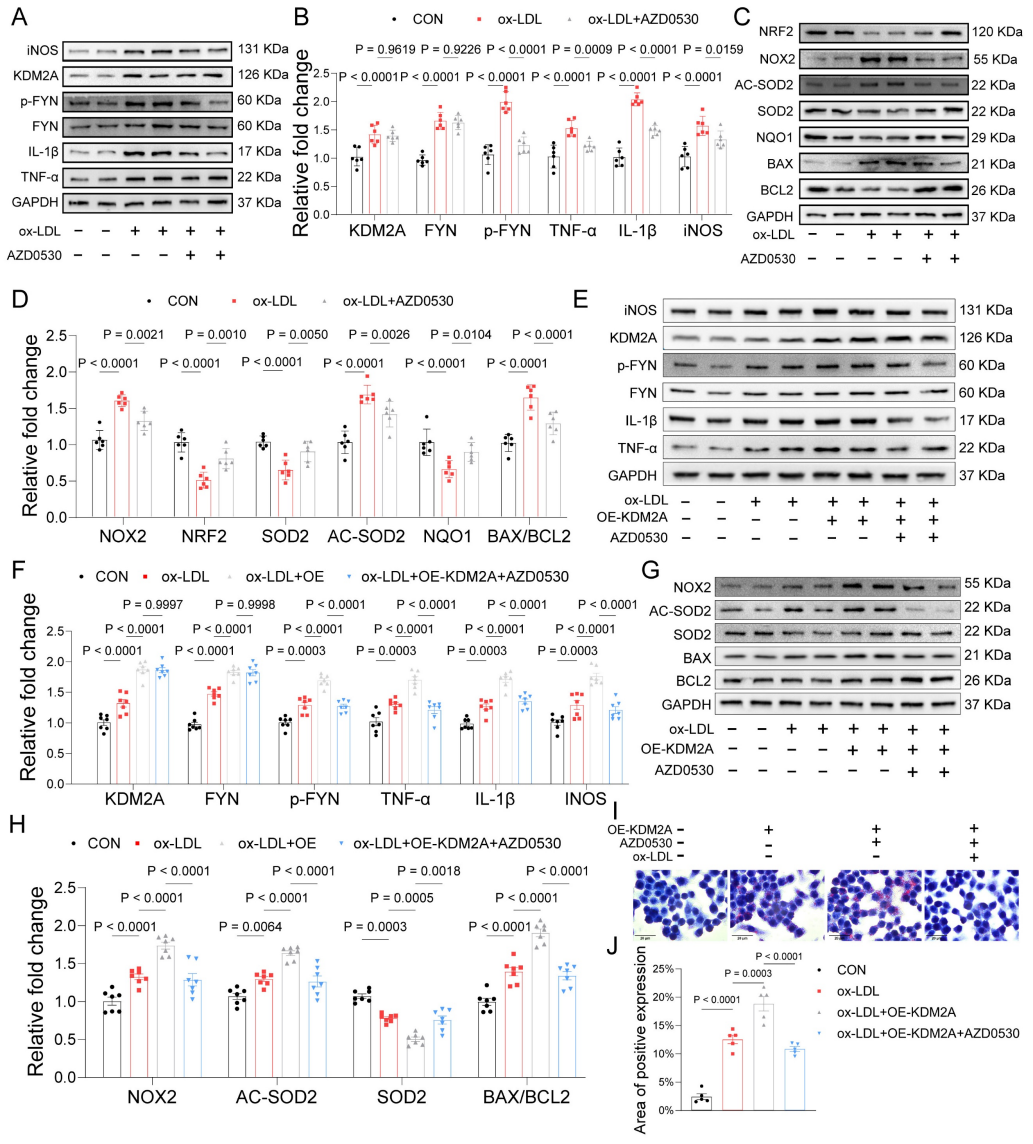
KDM2A-FYN axis exacerbates macrophage inflammatory response. (A-D) BMDMs were treated with the FYN activation inhibitor (AZD0530) for 24 hours after incubation with ox-LDL. Expression of inflammation (TNF-α, IL-1β, iNOS), oxidative stress (NRF2, NOX2, SOD2, NQO1, and AC-SOD2), and apoptosis (BAX and BCL2) was measured by immunoblotting and presented as bar plots together with group mean ± SEM (n = 6 per group). (E-H) BMDMs were treated with a KDM2A overexpression plasmid (OE-KDM2A) and rescued by AZD0530. The expression levels of inflammatory markers (TNF-α, IL-1β, iNOS), oxidative stress markers (NOX2, SOD2, and AC-SOD2), and apoptotic markers (BAX and BCL2) were measured by immunoblotting and presented as bar plots together with group mean ± SEM (n = 7 per group). (L) Oil-red O staining determined the lipid accumulation in RAW264.7 cells treated with OE-KDM2A and reversed by AZD0530, quantified by (M) bar plot (n=5 per group). The expressions were plotted together with the group mean ± SEM. data were compared by one-way ANOVA test.

AZD0530, a selective inhibitor of FYN phosphorylation, was used to explore the pro-inflammatory role in ox-LDL induced macrophages. We found that AZD0530 induction did not alter the expression of KDM2A and FYN but significantly reversed the increased p-FYN (Figure 8A-B). Levels of inflammatory markers (TNF-α, IL-1β, and iNOS) exhibited a dramatic reduction after inhibition of FYN phosphorylation (Figure 8A-B). Recent evidence has suggested a link between FYN and the oxidative response, which plays a critical role in driving macrophage M1 phenotypic transformation, thereby contributing to the development of atherosclerosis 68-70.

*In vitro* analyses further validated that FYN upregulates NOX2 and negatively regulates NRF2, the key TF in maintaining redox homeostasis (Figure 8C-D). Consistently, its downstream antioxidant regulators, SOD2 and NQO1, were also inhibited by AZD0530. Additionally, the expression of AC-SOD2 and BAX/BCL2 was downregulated upon inhibition of FYN activation (Figure 8C-D). Therefore, we presumed that KDM2A induces inflammatory response and oxidative stress via transcriptional activation of FYN in macrophages. Furthermore, overexpression of KDM2A in macrophages treated with ox-LDL exacerbated the expression of inflammatory markers (TNF-α, IL-1β, and iNOS), oxidative factors (NOX2, SOD2, and AC-SOD2) and apoptotic factors (BAX and BCL2). Conversely, inhibition of FYN largely rescued the functional changes in macrophages incubated with ox-LDL and the OE-KDM2A plasmid (Figure 8E-H). Oil-red O staining images revealed a significant increase in intracellular lipid droplet accumulation in macrophages after KDM2A overexpression, whereas the accumulation decreased in response to AZD0530 induction (Figure 8I-J).

### Macrophage KDM2A knockdown prevents atherosclerosis progression

**Figure 9:**
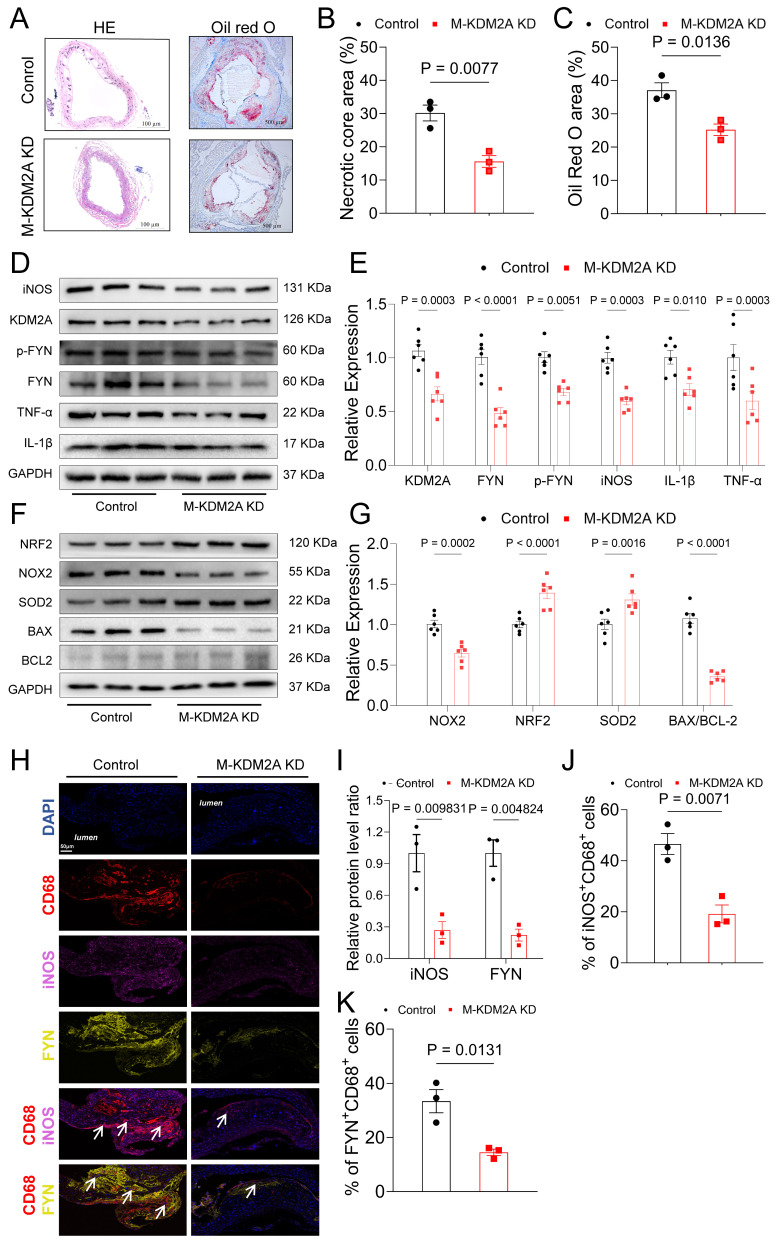
Inhibition of Macrophage KDM2A attenuates atherosclerotic progression. (A) HE staining of carotid arteries in ApoE^-/-^ mice treated with adeno-associated virus serotype 9 (AAV9) of negative control (HBAAV2/9-F4/80-mcherry, Control) or si-KDM2A (HBAAV2/9-F4/80-mir30-m-KDM2A-mcherry, M-KDM2A KD). (B) Quantitative analysis of the area of necrotic core (upper panel) and atherosclerotic lesion (lower panel) in aortic roots (n=3 per group). (C) Oil-red O staining of aortic roots from ApoE^-/-^ mice treated with AAV-NC or AAV-si-KDM2A. (D-G) Aortic tissue samples from control and M-KDM2A KD group were used to validate the dysregulation of KDM2A, p-FYN/FYN ratios, and associated pathways The expression levels of inflammatory markers (TNF-α, IL-1β, iNOS), oxidative stress markers (NRF2, NOX2, SOD2), and apoptosis-related proteins (BAX and BCL2) were assessed by immunoblotting and presented as bar plots (n=6 per group). (I) Representative immunofluorescence analysis of cross-sections of the aortic root stained with iNOS, FYN, and CD68. Quantification of the relative intensity iNOS/FYN (I) and percentages of iNOS^+^ (J) and FYN^+^ (K) macrophages (CD68^+^ cells). Values are presented as group mean ± SEM. Data were compared by Mann-Whitney U-test.

To verify the atherogenic role of KDM2A in vivo, we generated macrophage-specific KDM2A knockdown mice using AAV9-F4/80-si-KDM2A (M-KDM2A KD) via tail vein injection in ApoE^-/-^ mice. This strategy facilitated the specific knockdown of KDM2A in macrophages, as it encoded KDM2A siRNA under the control of the macrophage-specific marker (F4/80) expression (Supplementary figure 9). Macrophage knockdown of KDM2A significantly alleviated the endothelial swelling in the carotid artery (Figure 9A). Oil-Red O staining of aortic roots further demonstrated that macrophage KDM2A knockdown reduced the atherosclerotic lesion area and necrotic area (Figure 9A-C). Immunodetection in aortic root revealed a significant reduction in KDM2A expression within CD68^+^ cells by AAV9-F4/80-si-KDM2A (Supplementary figure 10A-C). Consistently, WB analysis confirmed the downregulation of KDM2A, p-FYN, FYN, and associated inflammatory and oxidative stress markers in aortic tissue from the M-KDM2A KD group (Figure 9D-G). The decrease in KDM2A^+^ macrophage proportion within the plaque of M-KDM2A-KO group was accompanied by reduced inflammation (iNOS^+^CD68^+^ cells), as well as its direct target FYN content (FYN^+^CD68^+^ cells) (Figure 9H-K). These findings indicated that the inhibition of KDM2A in macrophages reduced the progression of atherosclerosis by reducing macrophage inflammatory response and FYN upregulation.

### KDM2A genetically causes atherosclerosis and intervention compounds

**Figure 10:**
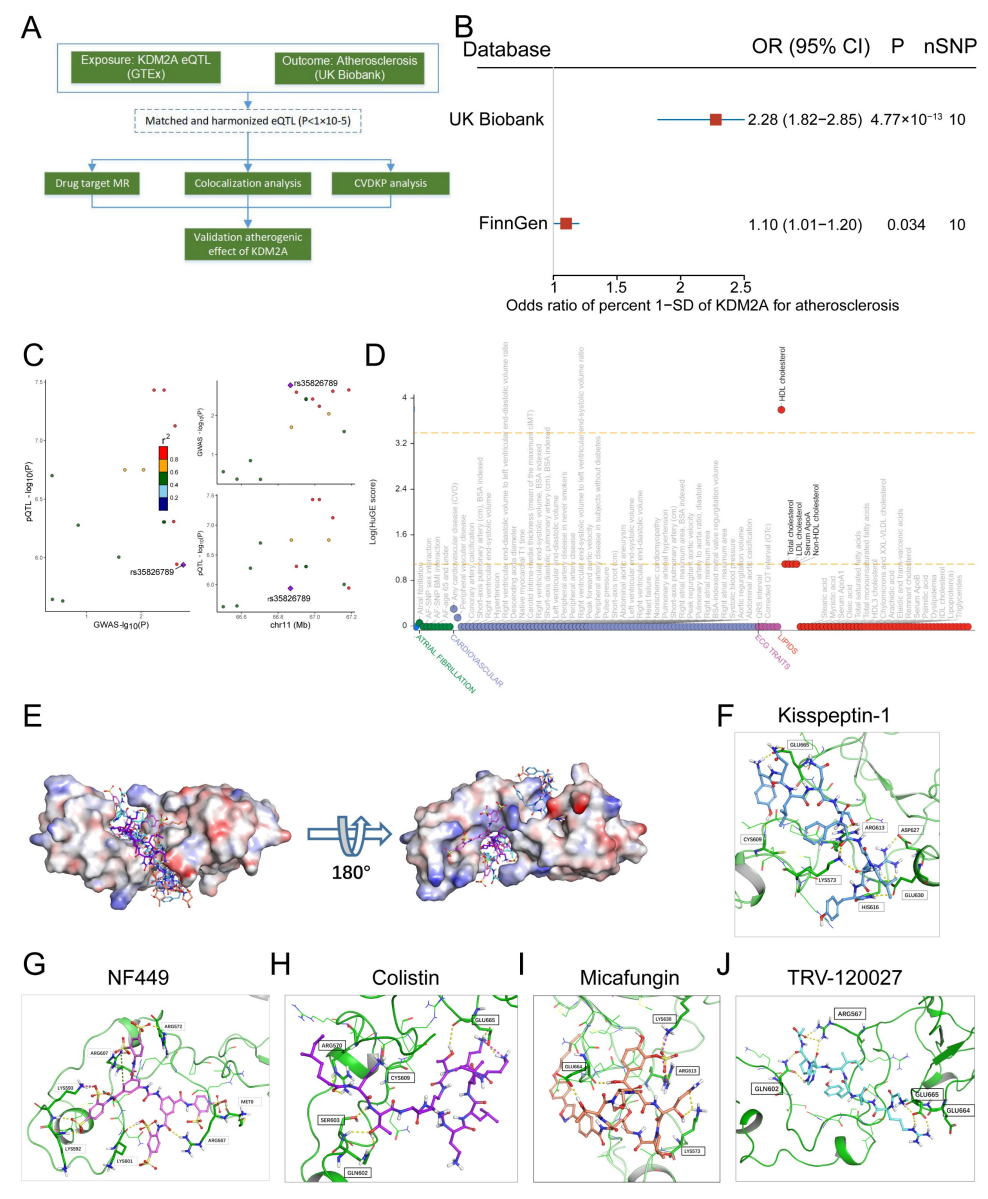
KDM2A casually predicts atherosclerosis. (A) The study flowchart shows the genetic association analysis of KDM2A with atherosclerosis; (B) Forest plot illustrating the association of KDM2A with risk of atherosclerosis using the random effects inverse-variance weighted (IVW) method across different databases (UK Biobank and FinnGen). (C) Regional association plot for the colocalization analysis of KDM2A expression with atherosclerosis risk, with the lead SNP shown as a purple diamond. (D) HuGE (Human Genetic Evidence) scores quantify the genetic support for the involvement of KDM2A in various diseases and traits available in the CVDKP platform. (E) Electrostatic potential diagram of KDM2A (PDB ID: 4BBQ) interacting with the five good candidates (Kisspeptin-1: blue, NF-449: pink, Colistin: purple, Micafungin: orange, and TRV-120027: cyan). Predicted binding models of Kisspeptin-1 (F), NF-449 (G), Colistin (H), Micafungin (I), and TRV-120027 (J) with the Zinc finger of KDM2A. KDM2A was shown as a green cartoon, and the critical residues in binding were colored in red. The five compounds were shown as pink sticks. Yellow dashed lines represented the potential hydrogen bonds.

The study flowchart for identifying a genetic association between KDM2A and atherosclerosis is shown in Figure 10A. Using the IVW method, genetically predicted higher levels of KDM2A were suggestively associated with atherosclerosis (Figure 10B). Additionally, sensitivity analysis demonstrated a robust genetic relationship (Supplementary figure 11A-B, Supplementary table 22-23). To assess whether KDM2A expression and atherosclerosis were consistently sharing the true casual variant instead of confounding of linkage disequilibrium, KDM2A was supported by suggestive evidence (PPH4 > 50%) through colocalization analysis, indicating promising probability for a shared casual variant between gene expression and atherosclerosis risk (Figure 10C and Supplementary table 24). We also investigated the potential effect of KDM2A on other cardiovascular phenotypes (such as lipids, atrial fibrillation, etc.). The strong association of HDL cholesterol with KDM2A and moderate associations of other lipid parameters (total cholesterol, LDL cholesterol, serum apolipoprotein A, and non-HDL cholesterol) were observed in the CVDKP platform (Figure 10D).

Through virtual screening, the top 5 small molecules (Kisspeptin-1, NF-449, Colistin, Micafungin, and TRV-120027) that directly target KDM2A based on its 3-dimensional structure were selected according to the docking score (Figure 10E and Supplementary table 25). According to the docking structure, all of the 5 compounds are predicted to bind the CXXC-type and PHD-type domain of KDM2A (Figure 10F-J and Supplementary figure 11C-G).

## Discussion

In this study, we reported for the first time the detrimental role of KDM2A in the development and progression of atherosclerosis through an unbiased screening pipeline. KDM2A induces abnormal lipid metabolism, oxidative stress, and inflammatory response in macrophages by activating FYN transcription. The pro-atherogenic role of KDM2A in macrophages has also been validated by *in vivo* studies (Figure 10).

Transcriptional activation is the initial process in cell phenotypic alteration, in which transcriptional regulators play a critical role. Previous art-of-review summarized several TFs that had been found to be associated with phenotypic alteration of macrophages in atherosclerosis 12. However, these TFs showed less specificity among different diseases and cell types, making them difficult to define as therapeutic targets. We first explored TRs during the development of atherosclerosis by constructing regulatory networks with regulon activity. With the advancement of scRNA-seq technology, we had uncovered that KDM2A is specifically involved in altering the functional state of macrophage. Additionally, we had also excluded the potential effect of KDM2A in other cell types (including T cells, VSMCs, and HUVECs). To date, there has been limited research establishing the association between KDM2A and macrophages. Longmin et al. showed that myeloid Kdm2a^-/-^ mice were protected from HFD-induced injury (including obesity, insulin resistance, hepatic steatosis, and reduced macrophage accumulation in adipose), the underlying mechanism attributed to increased H3K36me2 levels of Pparg and subsequently M2 polarization by Kdm2a knockout 18. However, the difference should be noted in our study. Kdm2a^-/-^ BMDMs showed unaltered in M1-program related gens (*Il1b* and *Nos2*) by RT-qPCR under lipopolysaccharide treatment in their study. While, we observed an obvious downregulation of pro-inflammatory markers (*Il1b*, *Tnfa*, *Nos2*) after Kdm2a knockdown after ox-LDL stimulation (Figure 4E-F), which revealed the specific role of KDM2A in macrophages during atherosclerosis. Furthermore, they focus on the rewiring effect of KDM2A on M2 macrophage transition instead of M1 reprogramming. We found the novel regulatory role of KDM2A in oxidative stress, and apoptosis under ox-LDL treatment, not only in the inflammatory response. Therefore, we thought that our study may shed the light on the novel regulatory effect of KDM2A on atherosclerosis.

Macrophage inflammatory response is persistent and plays a critical pathogenic role from plaque formation, growth and to rupture 3, 71-73. As macrophages are recruited from peripheral blood and undergo locally proliferation, the uptake of lipoproteins is typically recognized as the initial process of the inflammatory response 74-76. Upon stimulation with lipoproteins (such as ox-LDL), metabolic reprogramming can induce the secretion of cytokines and chemokines 77, 78. For instance, signals from CD36-mediated recognition with ox-LDL transduce to Toll-like receptor (TLR) heterodimers, specifically TLR2/4, leading to the formation of a heterotrimeric complex (CD36/TLR2/4) and activation of TLR signaling 79. Moreover, increased cellular cholesterol, characterized by enhanced cholesterol uptake via LDL receptor (LDLR) and reduced cholesterol efflux via ATP-binding cassette transporters ABCA1 and ABCG1, can activate NLRP3 inflammasome assembly and subsequent secretion of IL-1 family cytokines 80, 81. Excessive ROS production is another key feature of the macrophage inflammatory response to ox-LDL incubation 48, 82. ROS further promote the mitochondrial DNA (mtDNA) damage and release, as well as activation of pro-inflammatory transcription factors (such as NF-κB) in macrophages and necrotic core formation 63, 83, 84. Interestingly, we found that KDM2A not only regulates cytokines and chemokines secretion (IL-1β, TNF-α, etc.), but also influences lipid uptake (CD36 and LDLR), cholesterol efflux (ABCA1 and ABCG1), and oxidative stress (NOX2 and NRF2) in macrophages. And the underlying mechanism can be partly explained by FYN. FYN is a member of the Src family of protein kinases 85. FYN, primarily localized in cytoplasmic leaflets, facilitates the transport of various cell surface receptors (such as CD36) from the cytoplasm, thereby inducing an inflammatory response in macrophages 86, 87. In addition, a recent study shown that Fyn phosphonates c-Cbl at Tyr^731^, triggering the polyubiquitination of Sirt1 and subsequently increasing intracellular ROS levels in glomerular mesangial cells 88. Elisabetta's study also observed increased activation of Nrf2 in Fyn^-/-^ mouse erythroblasts compared to WT cells, although the underlying mechanism remains unclear 69. In conclusion, our finding suggests that KDM2A regulates phenotypic alterations in macrophages through the transcriptional activation of FYN during the development of atherosclerosis.

Anti-inflammation is increasingly recognized as a promising approach to atherosclerosis prevention, with agents such as canakinumab (IL-1β inhibitor) and sodium-glucose co-transporter-2 inhibitors, though their anti-inflammatory effects 89-92. However, careful evaluation of potential target proteins remains crucial, particularly when considering novel targets that play a significant role in atherosclerosis. In our study, we identified several small molecules as potential candidate for targeting KDM2A in the context of atherosclerosis. For instance, NF449 serves as a P2X1 receptor antagonist, which prevents fibrin generation and thrombus formation in previous study 93. Our findings suggest its potential role as a KDM2A antagonist in treatment for patients with CAD.

Although we identified a novel transcriptional regulator KDM2A involved in macrophage inflammatory response and atherosclerosis, several limitations should be noted. Firstly, incorporation of FYN macrophage-specific overexpression mice can further rescue atherogenic and inflammatory role of KDM2A. Secondly, the efficacy and safety of potential KDM2A-targeting compounds must be thoroughly assessed through *in vivo* experiments.

## Supplementary Material

Supplementary figures.

Supplementary tables.

## Figures and Tables

**Figure 11:**
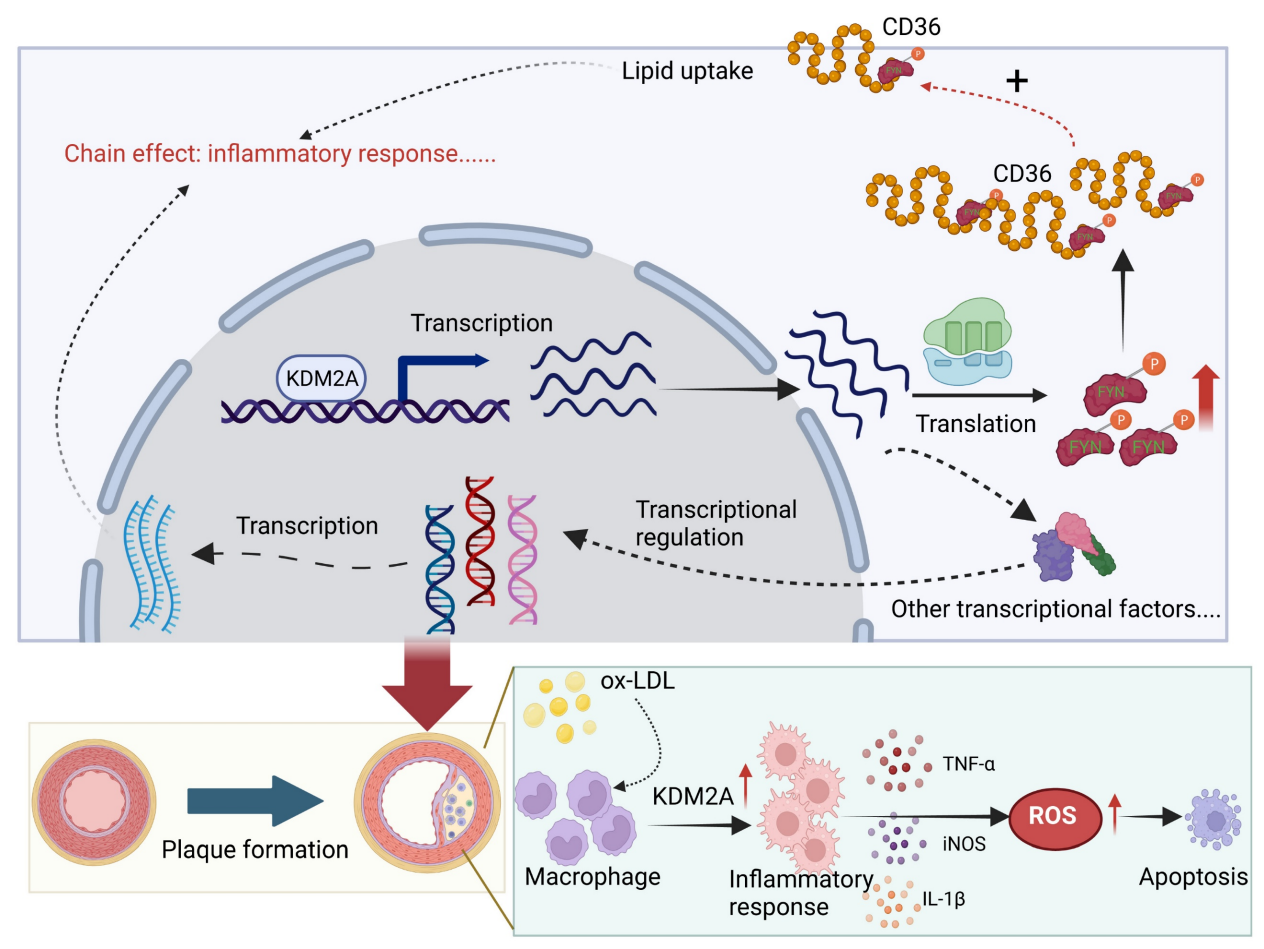
Schematic illustration of the role of KDM2A in the macrophage inflammatory response during atherosclerosis development. Graphic summary was generated with BioRender (https://app.biorender.com/).

**Table 1:** PCR primers used in this study

Use	Genes	Directions	Sequence (5' to 3')
**RT-qPCR**			
	Kdm2a	Forward	GGTCAATAGGCTGCCAGGATTAA
		Reverse	TCGATTGTCTTGACCTGGCTTAT
	Fyn	Forward	CGGGCATTGTACGACAACACT
		Reverse	AGCCTGGAAGTCCCGATAGA
	Rreb1	Forward	AGCCTGGAAGTCCCGATAGA
		Reverse	GTAGGGCTTCTCGCCCGTAT
	Bcl2l11	Forward	AAATGGCCAAGCAACCTTCTG
		Reverse	CTTGCGGTTCTGTCTGTAGGG
	Runx1	Forward	TGAAGAACCAGGTAGCGAGATTC
		Reverse	GCAACTTGTGGCGGATTTGTAAA
	Arid1a	Forward	CTCTGGACCTCTATCGCCTCTAT
		Reverse	CACATTGAGGTTGGTTGCAAGTT
	Rnf213	Forward	TGACCTCCGCAGATCAACAATTA
		Reverse	TCCATTTGTTCCTCTGCCAATTC
	Mafb	Forward	TTGAGGGAAAGCTAAGGGAGAGA
		Reverse	CGTGGGTGTGTGTATGTCAATTT
	Aff1	Forward	GAAGGAAAGACGCAACCAAGA
		Reverse	TAGCTCATCGCCTTTTGCAGT
**ChIP-qPCR**			
	Fyn-1	Forward	GGCGTCCCTTTCTTTCTGGT
		Reverse	ACAGCTAAATGCCTGCACCT
	Fyn-2	Forward	ACTGGTAGCATTGAGTGGCA
		Reverse	GGGGAAATGAGGGATAGGGG
