# Supplementary material for: Macrophage KDM2A promotes atherosclerosis via regulating FYN and inducing inflammatory response
Source: Int J Biol Sci. 2025 Mar 31;21(6):2780–805. doi: 10.7150/ijbs.102675 (PMC12035892; doi:10.7150/ijbs.102675)
Supplement: Supplementary file 1 — Supplementary figures. [file ijbsv21p2780s1.pdf]

Supplementary Figure legends

Supplementary figure 1: Validation of upregulated expression of KDM2A in plaque. (A) Differentially expressed regulons identified between atherosclerotic plaque and macroscopically intact tissue samples in GSE43292 (P adj < 0.05 and |Fold change| > 2). (B) Heatmap showing the top 10 activated and deactivated transcriptional regulators (TRs) in the plaque group. (C) Network displaying three master regulons and their target genes (red line: positive correlation; blue line: negative correlation). (D) Boxplots together with group mean ± SE illustrating the activities of the three TRs (DLX2, HESX1, and KDM2A) that were up- or down-regulated in the plaque group based on the combine dataset (n = 235 in total, AS=195, normal=40). (E) Transcriptional expression of the three TRs (DLX2, HESX1, and KDM2A) between early (n=13) and advanced (n=16) atherosclerotic plaque groups in GSE28829. Correlation analysis of coronary artery disease (CAD) class with (F) DLX2 and (G) HESX1 in GSE90074. (H) The difference of the three TRs (DLX2, HESX1, and KDM2A) levels between the non-obstructive (n=50) and obstructive (n=93) CAD groups. (I) Binary regression analysis shows the significant correlation between KDM2A levels and obstructive CAD, adjusting for demographic characteristics (age, sex, diabetes, hypertension, and hyperlipidemia). (J) Enrichment analysis of KDM2A target genes. Correlation analysis was performed using the Spearman's test; for comparisons two groups, data were assessed by Wilcoxon signed-rank test: ns: $P > 0.05$, *$P \leq 0.05$, **$P < 0.01$. Expressions were presented in boxplots together with the group mean ± SE.

Supplementary figure 2: Inference of immune infiltration revealed the association of KDM2A with macrophage subtypes. (A) Correlation of the three TRs (HESX1, DLX2, and KDM2A) with 28 immune cell types by single sample gene set enrichment analysis (ssGSEA) in the combine dataset. (B) Boxplots show the differences in the proportions of different macrophage subtypes between macroscopically intact (n=40) and atheroma plaque tissue (n=195) groups based on the combine dataset. (C) The correlation of the three TRs (HESX1, DLX2, and KDM2A) with different macrophage subtypes through four different algorithms (ssGSEA, CIBERSORT, MCP-counter, and xCell) in the combine dataset. Boxplots show the differences in the proportions of different T cell subtypes between macroscopically intact and atheroma plaque tissue group based on (D) GSE43292 and (E) combine dataset. Correlation of the three TRs (HESX1, DLX2, and KDM2A) with different T cell subtypes using four different algorithms (ssGSEA, CIBERSORT, MCP-counter, and xCell) in (F) GSE43292 and (G) combine dataset. Correlation analysis was examined by Spearman's test; for two groups, data were compared by Wilcoxon signed-rank test: *$P < 0.05$, **$P < 0.01$, ***$P < 0.001$, ****$P < 0.0001$. Expressions were presented as boxplots together with the group mean ± SE.

Supplementary figure 3: KDM2A exhibits a higher specificity in macrophages. (A) Analysis of the relative expression of KDM2A across various organs based on the data from the GTEx database shown as box/violin plots. (B) t-SNE clustering plots showing a 19 color-coded cell clusters between proximal adjacent (PA) and paired

atherosclerotic core (AC) regions from 3 patient carotid arteries in GSE159677. (C) Violin plot showcasing the top marker gens across all cell types along with their respective cell proportions. (D) Examination of the relative expression levels of KDM2A across different main cell types shown as box/violin plots, as determined by data from the GSE159677 dataset. Data were statistically compared using the Wilcoxon signed-rank test.

Supplementary figure 4: Involvement of KDM2A in macrophage state alterations using single-cell RNA-seq data. (A) Violin plot displaying the expression levels of top marker genes in macrophage subtypes. (B) Bar chart showing the relative frequencies of macrophage subtypes in proximal adjacent (PA) and paired atherosclerotic core (AC) groups. Single-cell trajectories of the macrophages represented through (C) pseudotime, (D) different cell types, (E) different states, and (F) PA and AC groups as analyzed by Monocle. (G) Bar chart showing the relative frequency of macrophage states in PA and AC groups. (H) The difference in KDM2A activity observed across different macrophage states shown as box/violin plots. Data were presented with group mean $\pm$ SE and compared by Wilcoxon signed-rank test. (I) Heatmap showing the expression of inflammatory macrophage and neutrophil markers across different macrophage subtypes. (J) Immunofluorescence illustrating the cells expressing KDM2A co-expressed with Ly6G within the mouse plaque lesions.

Supplementary figure 5: Validation of KDM2A's involvement in macrophage phenotypic transition using Smart-seq2 based scRNA-seq data (GSE260657). t-SNE clustering plots showing a (A) 10 cell populations (a total of 7,686 individual cells), (B) 12 cell clusters, and (C) two groups (asymptomatic and symptomatic groups). (D) Violin plot showcasing the top marker gens across all cell types. (E) Examination of the relative expression levels of KDM2A across different main cell types shown as violin plots. (F) Cluster tree of myeloid cells sub-clustering according to the different resolutions. t-SNE clustering plots showing a (G) 4 cell populations (a total of 2,900 individual cells), (H) 4 cell clusters, and (I) two groups. (I) Heatmap showing the expression of myeloid subtype markers across different clusters. (K) Examination of the relative expression levels of KDM2A across different myeloid subtypes shown as violin plots. (L) Displayed genes ordered by correlation coefficient with KDM2A activity.

Supplementary figure 6: KDM2A is specifically upregulated in ox-LDL treated macrophages. Western blot assays assessing the expression of KDM2A in human umbilical vein endothelial cells (HUVECs) cultured with (A) different concentrations of ox-LDL ($n = 3$ per group) and (B) at different time points ($n = 6$ per group). (C) Similarly, Western blot assay showing the expression of KDM2A in mouse vascular smooth muscle cells (VSMCs) cultured with different concentrations of ox-LDL ($n = 3$ per group). (D) Expressions of KDM2A were examined in bone marrow-derived macrophages (BMDMs) cultured with varying concentrations of ox-LDL ($n = 3$ per group). Changes in KDM2A expression in BMDMs cultured at different time points

were assessed at (E) protein levels (n = 6 per group) and (F) RNA (n = 3 per group). (G) KDM2A expression levels after treatment with three different KDM2A siRNAs (n = 3 per group). (H) Upregulation of potential KDM2A target genes rescued by si-KDM2A (n = 3 per group). Expressions were plotted as bar plots together with group mean ± SEM. Data were compared by Mann–Whitney U-test for two groups and one-way ANOVA test for more than two groups.

Supplementary figure 7: Correlation analysis of KDM2A with target genes. (A) Gene ontology (GO) analysis of KDM2A binding peaks is presented as a bar chart. Association analysis of KDM2A with (B) FYN and (C) RREB1 using the combine dataset (plaque group, n=195). The association of KDM2A with (D) FYN and (E) RREB1 expression was examined in the GTEx database. Correlation analysis was performed using Pearson test.

Supplementary figure 8: The KDM2A-FYN axis in peripheral monocytes/macrophages correlates with atherosclerotic severity. The association of (A) KDM2A and (B) FYN expression with carotid plaque severity. (C) Validation of the strong association of KDM2A with RREB1 in GSE56045. The association of RREB1 expression with (D) CAC score and (E) carotid plaque severity. (F) Correlation analysis between KDM2A expression and CpG sites (β values) in 1202 patients. DNA gel electrophoresis showing the relative abundance of (G) Fyn-1 and (H) Fyn-2 promoter fragments among Input, anti-rabbit IgG, and Immunoprecipitation groups. The correlation of the levels of (I) TNF-α, (J) IL-6, and (K) HMGB in serum and Gensini score validated by Enzyme-linked immunosorbent assay (ELISA). Correlation analysis was examined using Pearson's test.

Supplementary figure 9: The experimental flowchart for the AAV9-F4/80-si-KDM2A infection animal experiment.

Supplementary figure 10: (A) Representative immunofluorescence analysis of cross-sections of the aortic root stained with KDM2A and CD68. (B) Quantification of the relative intensity KDM2A (B) and percentages of KDM2A$^+$ macrophages (CD68$^+$ cells) (C). Values are presented as bar plots with group mean ± SEM. Data were compared by Mann-Whitney U-test.

Supplementary figure 11: Causal relationship between KDM2A and atherosclerosis. Leave-one-out plot of the causal relationship between KDM2A and atherosclerosis in (A) UK Biobank and (B) FinnGen database. Surface plot showed the predicted binding models of Kisspeptin-1 (C), NF-449 (D), Colistin (E), Micafungin (F), and TRV-120027 (C) with the Zinc finger of KDM2A.

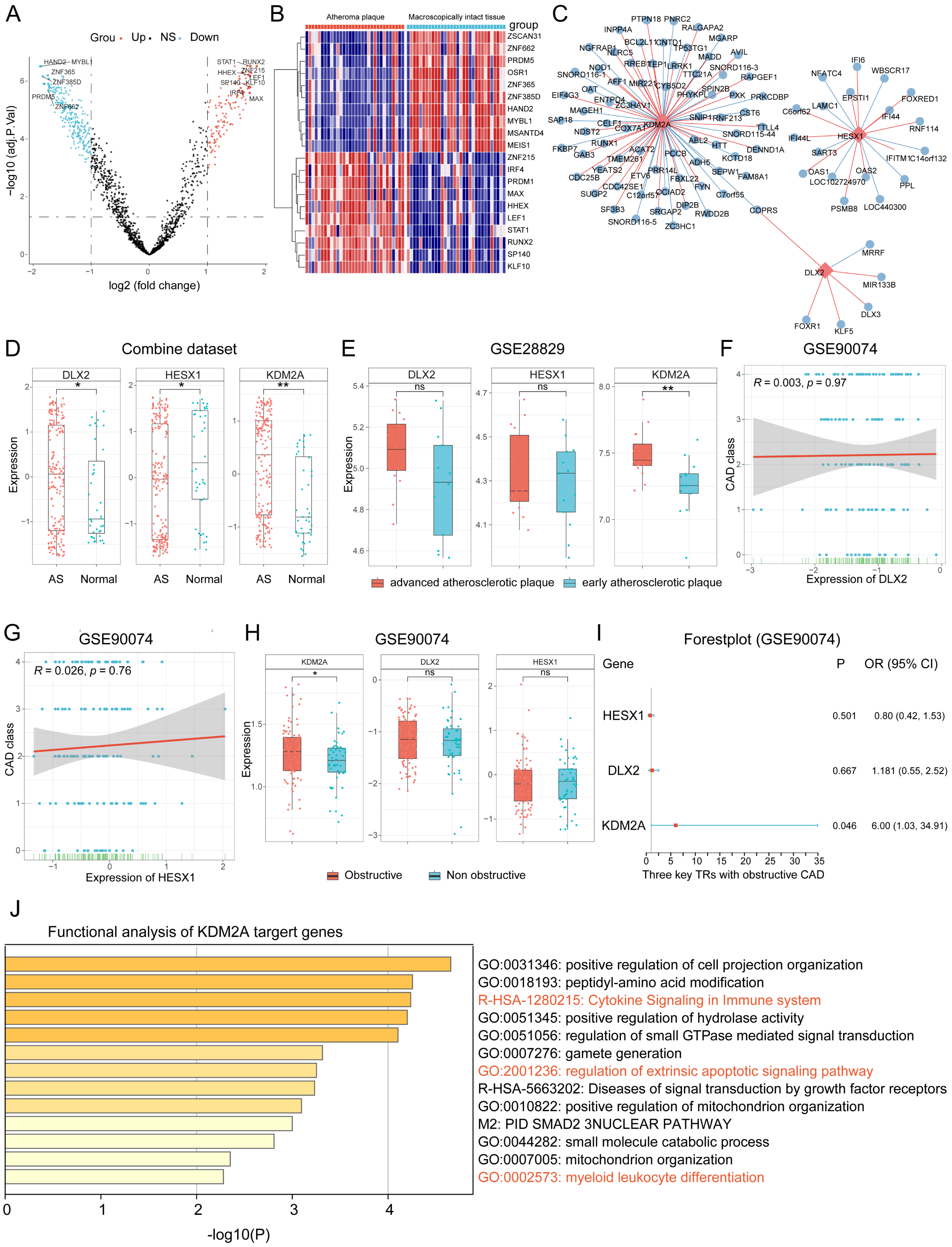
A
B
C
D
Combine dataset
E
GSE28829
F
GSE90074
G
GSE90074
H
GSE90074
I
Forestplot (GSE90074)
Gene P OR (95% CI)
HESX1 0.501 0.80 (0.42, 1.53)
DLX2 0.667 1.181 (0.55, 2.52)
KDM2A 0.046 6.00 (1.03, 34.91)
Three key TRs with obstructive CAD
advanced atherosclerotic plaque
early atherosclerotic plaque
Obstructive
Non obstructive
J
Functional analysis of KDM2A targert genes
GO:0031346: positive regulation of cell projection organization
GO:0018193: peptidyl-amino acid modification
R-HSA-1280215: Cytokine Signaling in Immune system
GO:0051345: positive regulation of hydrolase activity
GO:0051056: regulation of small GTPase mediated signal transduction
GO:0007276: gamete generation
GO:2001236: regulation of extrinsic apoptotic signaling pathway
R-HSA-5663202: Diseases of signal transduction by growth factor receptors
GO:0010822: positive regulation of mitochondrion organization
M2: PID SMAD2 3NUCLEAR PATHWAY
GO:0044282: small molecule catabolic process
GO:0007005: mitochondrion organization
GO:0002573: myeloid leukocyte differentiation
-log10(P)

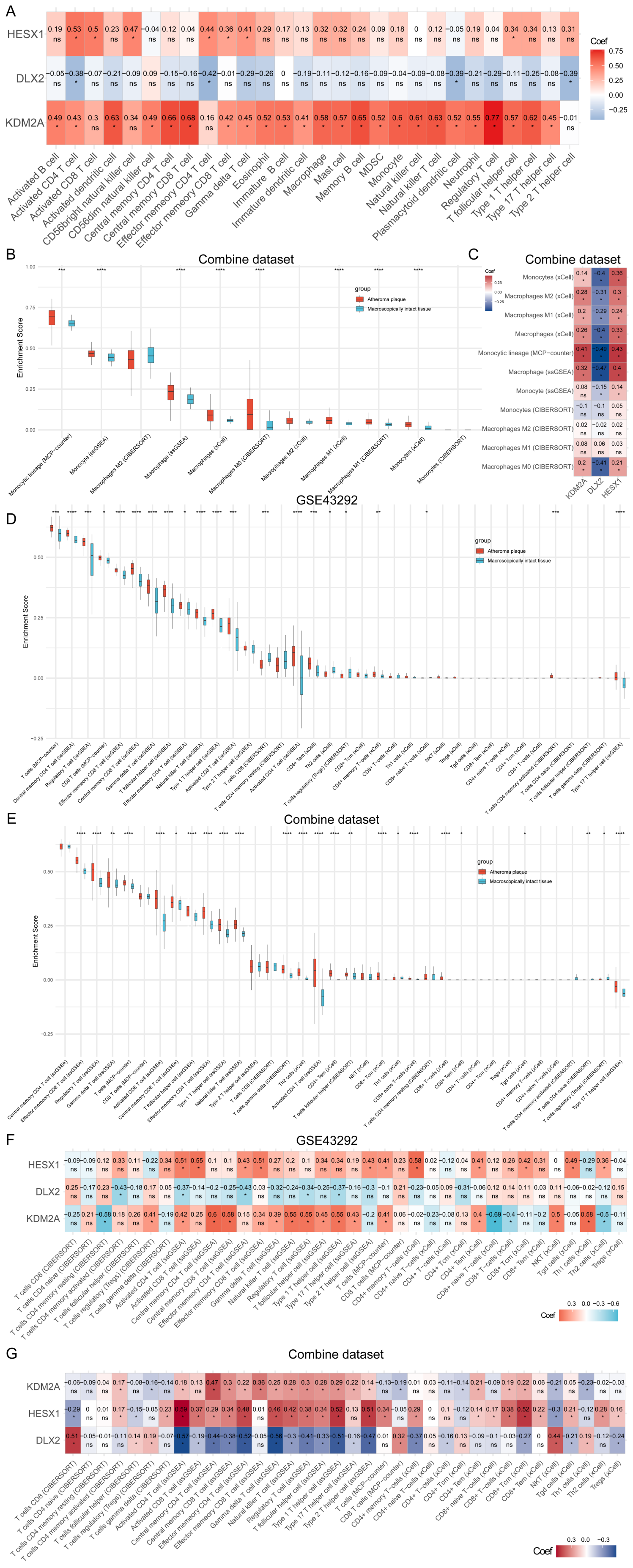
A
HESX1
DLX2
KDM2A
Coef
0.75
0.50
0.25
0.00
−0.25
Activated B cell
Activated CD4 T cell
Activated CD8 T cell
Activated dendritic cell
CD56bright natural killer cell
CD56dim natural killer cell
Central memory CD4 T cell
Central memory CD8 T cell
Effector memeory CD4 T cell
Effector memeory CD8 T cell
Gamma delta T cell
Eosinophil
Immature B cell
Immature dendritic cell
Macrophage
Mast cell
Memory B cell
MDSC
Monocyte
Natural killer cell
Natural killer T cell
Plasmacytoid dendritic cell
Neutrophil
Regulatory T cell
T follicular helper cell
Type 1 T helper cell
Type 17 T helper cell
Type 2 T helper cell
B
Combine dataset
Enrichment Score
group
Atheroma plaque
Macroscopically intact tissue
C
Combine dataset
Coef
KDM2A
DLX2
HESX1
D
GSE43292
Enrichment Score
group
Atheroma plaque
Macroscopically intact tissue
E
Combine dataset
Enrichment Score
group
Atheroma plaque
Macroscopically intact tissue
F
GSE43292
HESX1
DLX2
KDM2A
Coef
G
Combine dataset
KDM2A
HESX1
DLX2
Coef

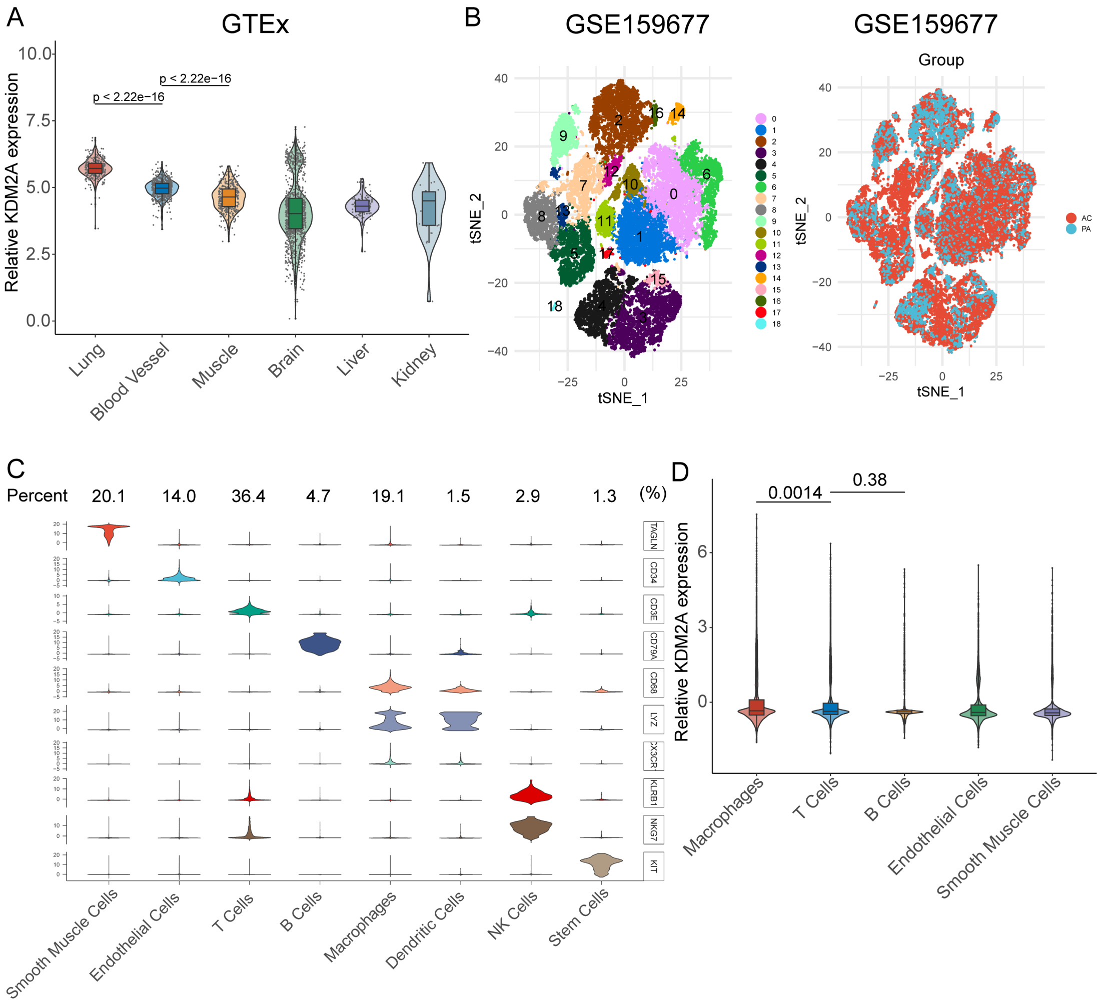
A
GTEx
Relative KDM2A expression
p < 2.22e−16
p < 2.22e−16
10.0
7.5
5.0
2.5
0.0
Lung
Blood Vessel
Muscle
Brain
Liver
Kidney
B
GSE159677
GSE159677
Group
tSNE_1
tSNE_2
AC
PA
C
Percent
20.1
14.0
36.4
4.7
19.1
1.5
2.9
1.3
(%)
TAGLN
CD34
CD3E
CD79A
CD68
LYZ
CX3CR1
KLRB1
NKG7
KIT
Smooth Muscle Cells
Endothelial Cells
T Cells
B Cells
Macrophages
Dendritic Cells
NK Cells
Stem Cells
D
0.0014
0.38
Relative KDM2A expression
Macrophages
T Cells
B Cells
Endothelial Cells
Smooth Muscle Cells

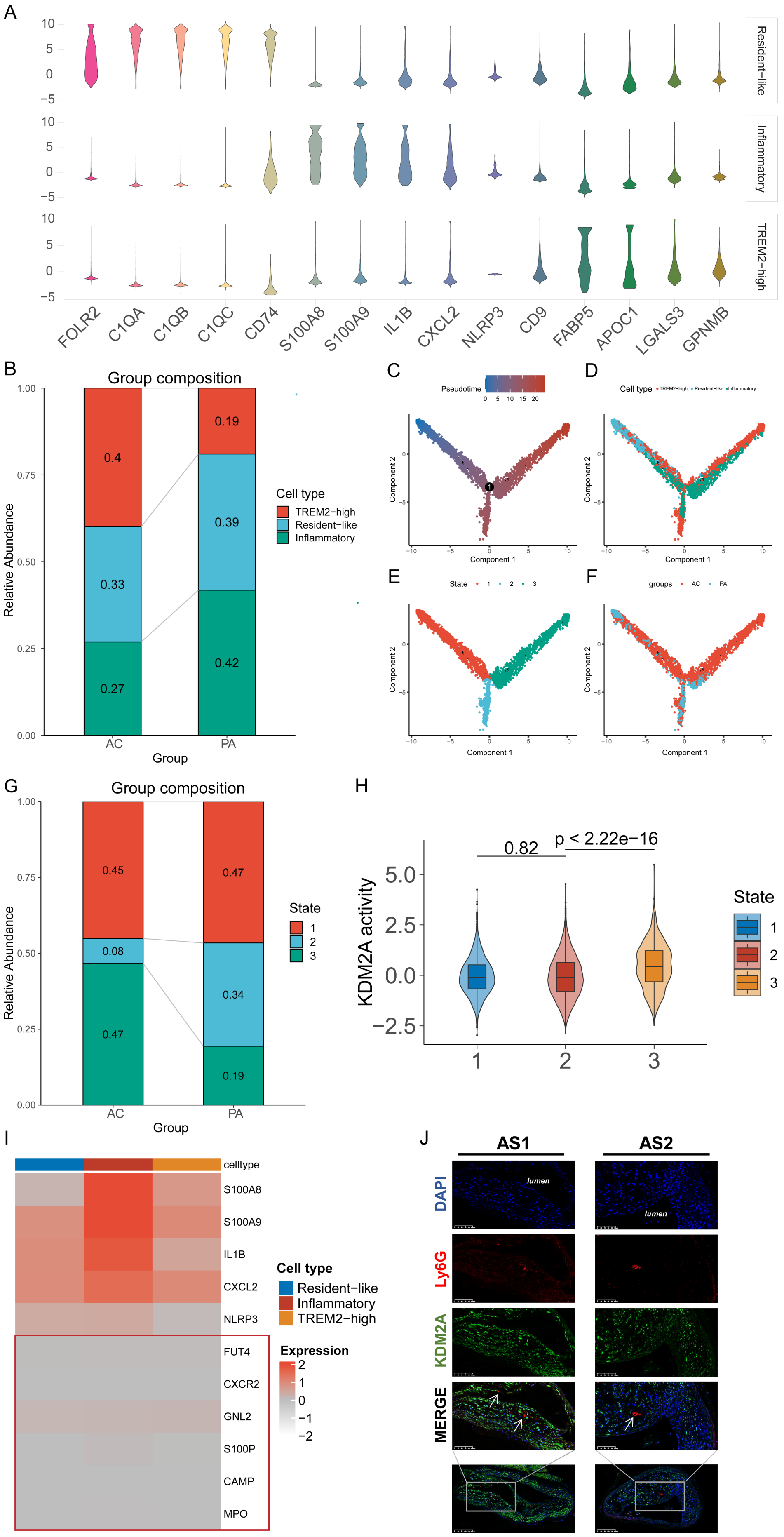

A
Resident−like
Inflammatory
TREM2−high
FOLR2
C1QA
C1QB
C1QC
CD74
S100A8
S100A9
IL1B
CXCL2
NLRP3
CD9
FABP5
APOC1
LGALS3
GPNMB
B
Group composition
Relative Abundance
Group
AC
PA
0.4
0.33
0.27
0.19
0.39
0.42
Cell type
TREM2−high
Resident−like
Inflammatory
C
Pseudotime
Component 1
Component 2
D
Cell type
E
State
F
groups
AC
PA
G
Group composition
Relative Abundance
Group
0.45
0.08
0.47
0.47
0.34
0.19
State
1
2
3
H
KDM2A activity
0.82
p < 2.22e−16
State
I
celltype
S100A8
S100A9
IL1B
CXCL2
NLRP3
FUT4
CXCR2
GNL2
S100P
CAMP
MPO
Cell type
Resident−like
Inflammatory
TREM2−high
Expression
J
AS1
AS2
DAPI
Ly6G
KDM2A
MERGE
lumen

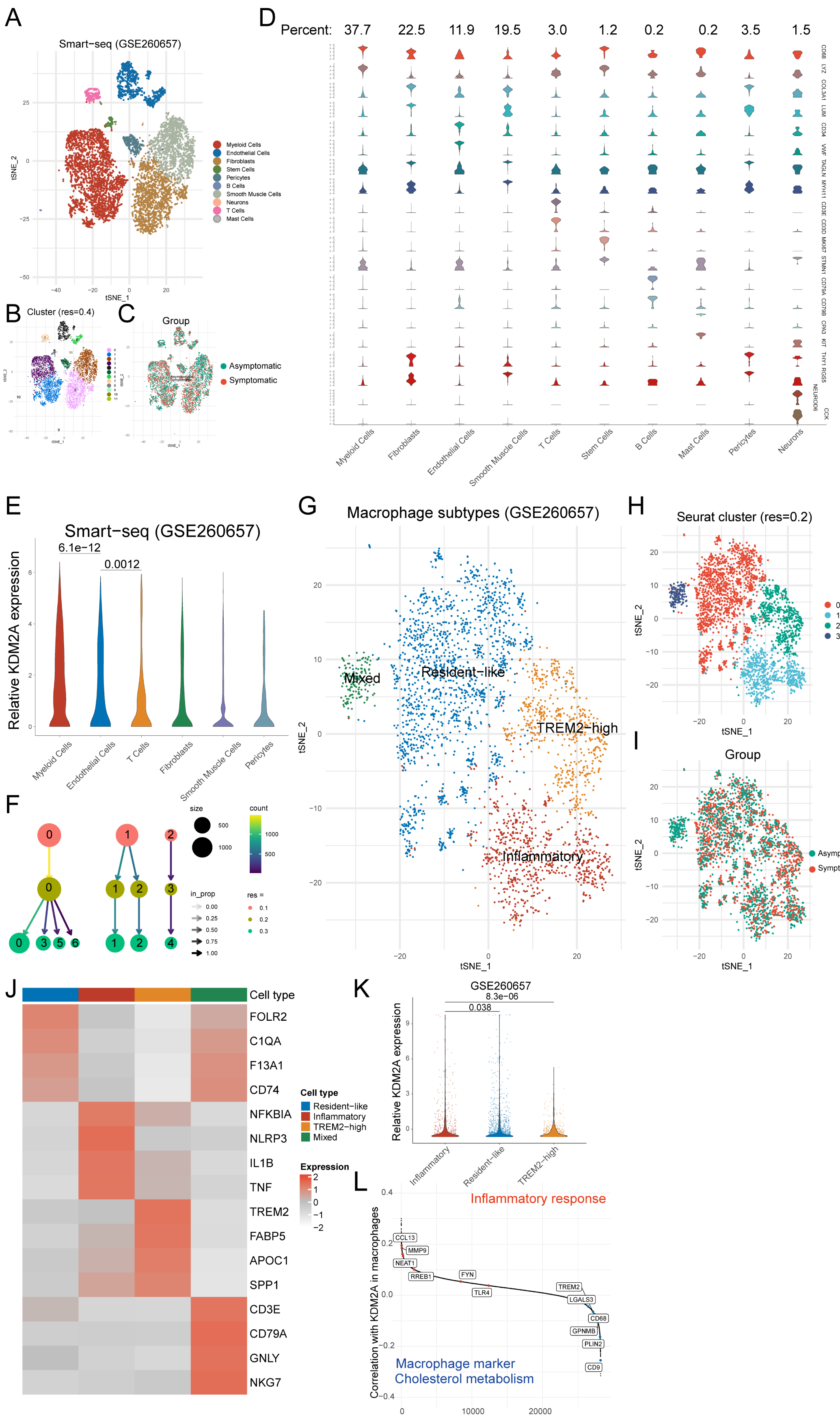

A
Smart−seq (GSE260657)
Myeloid Cells
Endothelial Cells
Fibroblasts
Stem Cells
Pericytes
B Cells
Smooth Muscle Cells
Neurons
T Cells
Mast Cells
tSNE_1
tSNE_2
B
Cluster (res=0.4)
C
Group
Asymptomatic
Symptomatic
D
Percent: 37.7 22.5 11.9 19.5 3.0 1.2 0.2 0.2 3.5 1.5
CD68 LYZ COL3A1 LUM CD34 VWF TAGLN MYH11 CD3E CD3D MKI67 STMN1 CD79A CD79B CPA3 KIT THY1 RGS5 NEUROD6 CCK
Myeloid Cells
Fibroblasts
Endothelial Cells
Smooth Muscle Cells
T Cells
Stem Cells
B Cells
Mast Cells
Pericytes
Neurons
E
Smart−seq (GSE260657)
6.1e−12
0.0012
Relative KDM2A expression
Myeloid Cells
Endothelial Cells
T Cells
Fibroblasts
Smooth Muscle Cells
Pericytes
F
size
count
in_prop
res =
G
Macrophage subtypes (GSE260657)
Mixed
Resident−like
TREM2−high
Inflammatory
tSNE_1
tSNE_2
H
Seurat cluster (res=0.2)
I
Group
Asymptomatic
Symptomatic
J
Cell type
FOLR2
C1QA
F13A1
CD74
NFKBIA
NLRP3
IL1B
TNF
TREM2
FABP5
APOC1
SPP1
CD3E
CD79A
GNLY
NKG7
Cell type
Resident−like
Inflammatory
TREM2−high
Mixed
Expression
K
GSE260657
8.3e−06
0.038
Relative KDM2A expression
Inflammatory
Resident−like
TREM2−high
L
Inflammatory response
Correlation with KDM2A in macrophages
CCL13
MMP9
NEAT1
RREB1
FYN
TLR4
TREM2
LGALS3
CD68
GPNMB
PLIN2
CD9
Macrophage marker
Cholesterol metabolism

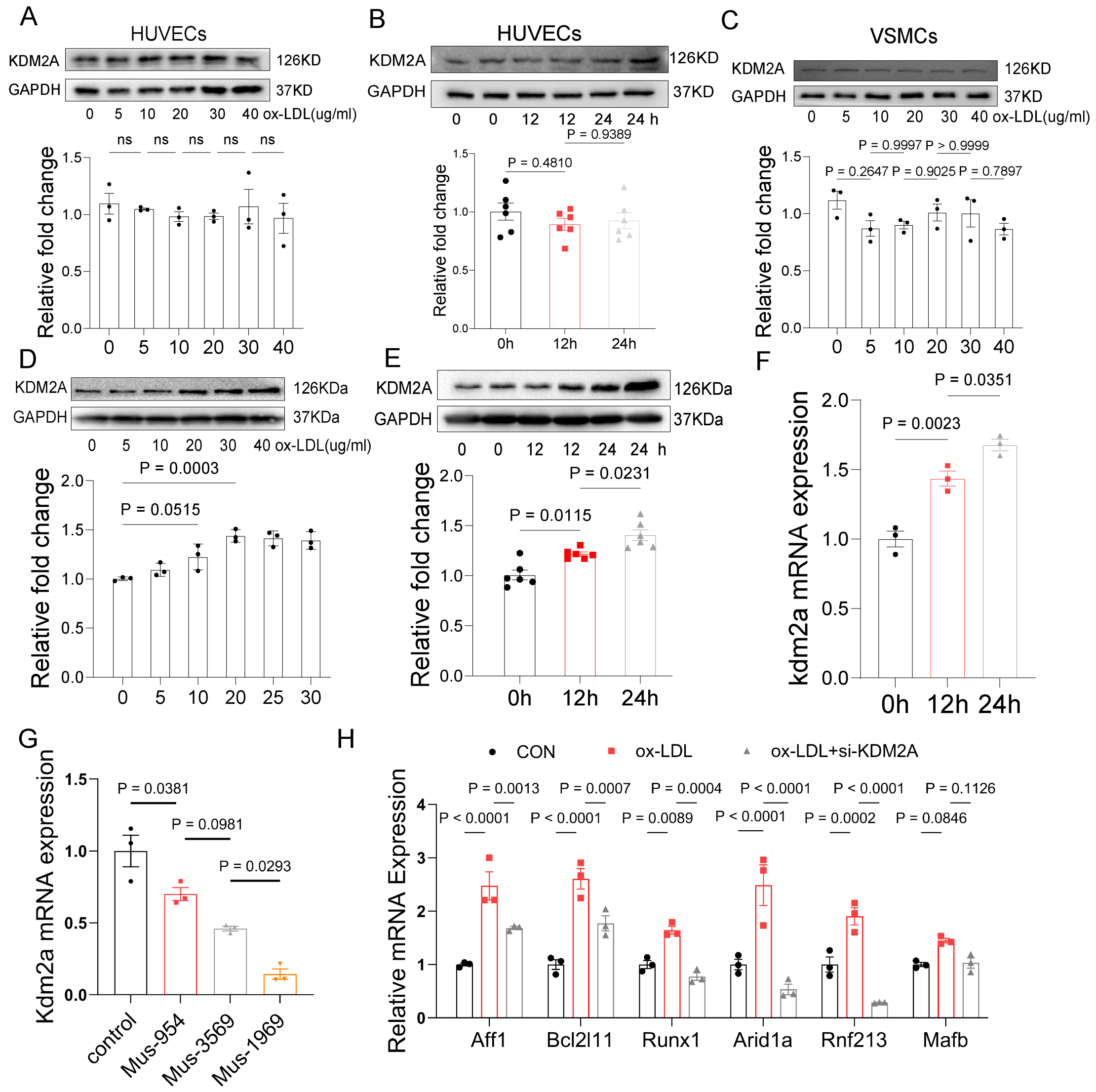

A
HUVECs
KDM2A
126KD
GAPDH
37KD
0 5 10 20 30 40 ox-LDL(ug/ml)
ns ns ns ns ns
Relative fold change
0 5 10 20 30 40
B
HUVECs
KDM2A
126KD
GAPDH
37KD
0 0 12 12 24 24 h
P = 0.9389
P = 0.4810
Relative fold change
0h 12h 24h
C
VSMCs
KDM2A
126KD
GAPDH
37KD
0 5 10 20 30 40 ox-LDL(ug/ml)
P = 0.9997 P > 0.9999
P = 0.2647 P = 0.9025 P = 0.7897
Relative fold change
0 5 10 20 30 40
D
KDM2A
126KDa
GAPDH
37KDa
0 5 10 20 30 40 ox-LDL(ug/ml)
P = 0.0003
P = 0.0515
Relative fold change
0 5 10 20 25 30
E
KDM2A
126KDa
GAPDH
37KDa
0 0 12 12 24 24 h
P = 0.0231
P = 0.0115
Relative fold change
0h 12h 24h
F
P = 0.0351
P = 0.0023
kdm2a mRNA expression
0h 12h 24h
G
P = 0.0381
P = 0.0981
P = 0.0293
Kdm2a mRNA expression
control
Mus-954
Mus-3569
Mus-1969
H
CON
ox-LDL
ox-LDL+si-KDM2A
P = 0.0013 P = 0.0007 P = 0.0004 P < 0.0001 P < 0.0001 P = 0.1126
P < 0.0001 P < 0.0001 P = 0.0089 P < 0.0001 P = 0.0002 P = 0.0846
Relative mRNA Expression
Aff1 Bcl2l11 Runx1 Arid1a Rnf213 Mafb

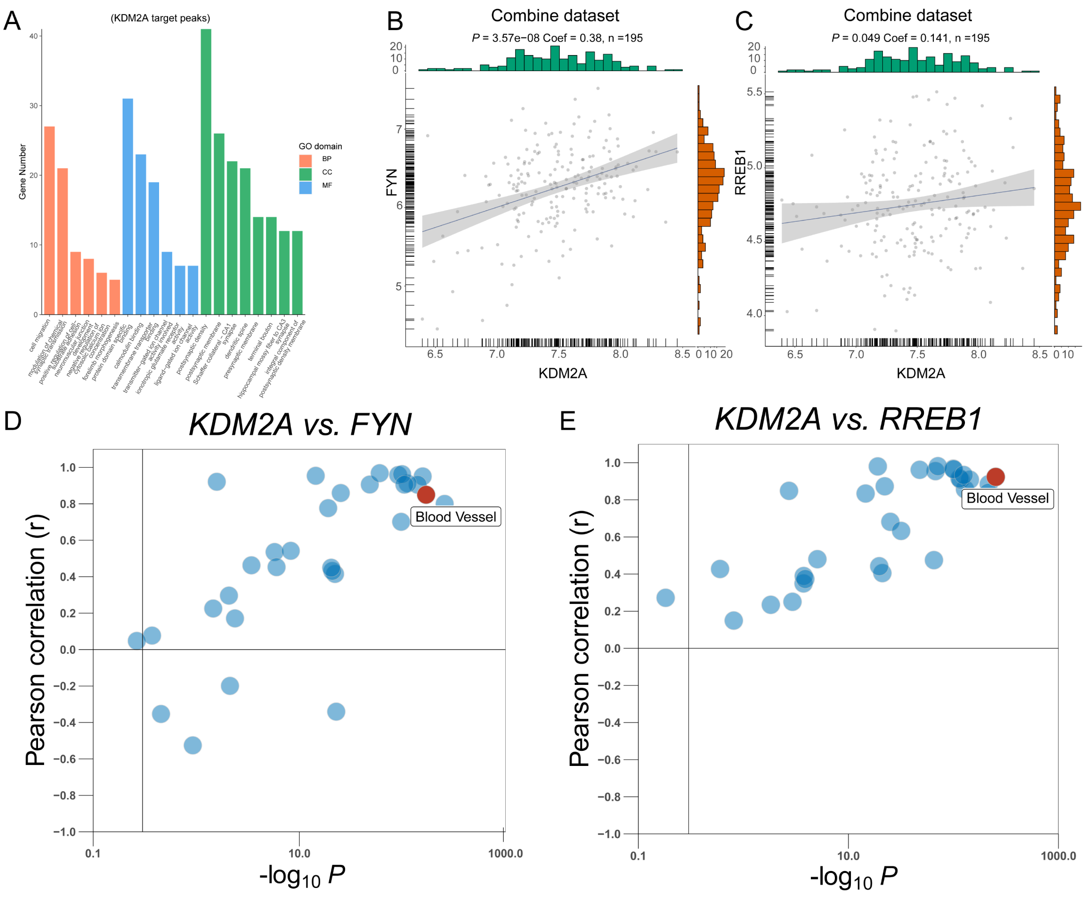
A
(KDM2A target peaks)
Gene Number
GO domain
BP
CC
MF
B
Combine dataset
P = 3.57e−08 Coef = 0.38, n =195
FYN
KDM2A
C
Combine dataset
P = 0.049 Coef = 0.141, n =195
RREB1
KDM2A
D
KDM2A vs. FYN
Pearson correlation (r)
Blood Vessel
-log10 P
E
KDM2A vs. RREB1
Pearson correlation (r)
Blood Vessel
-log10 P

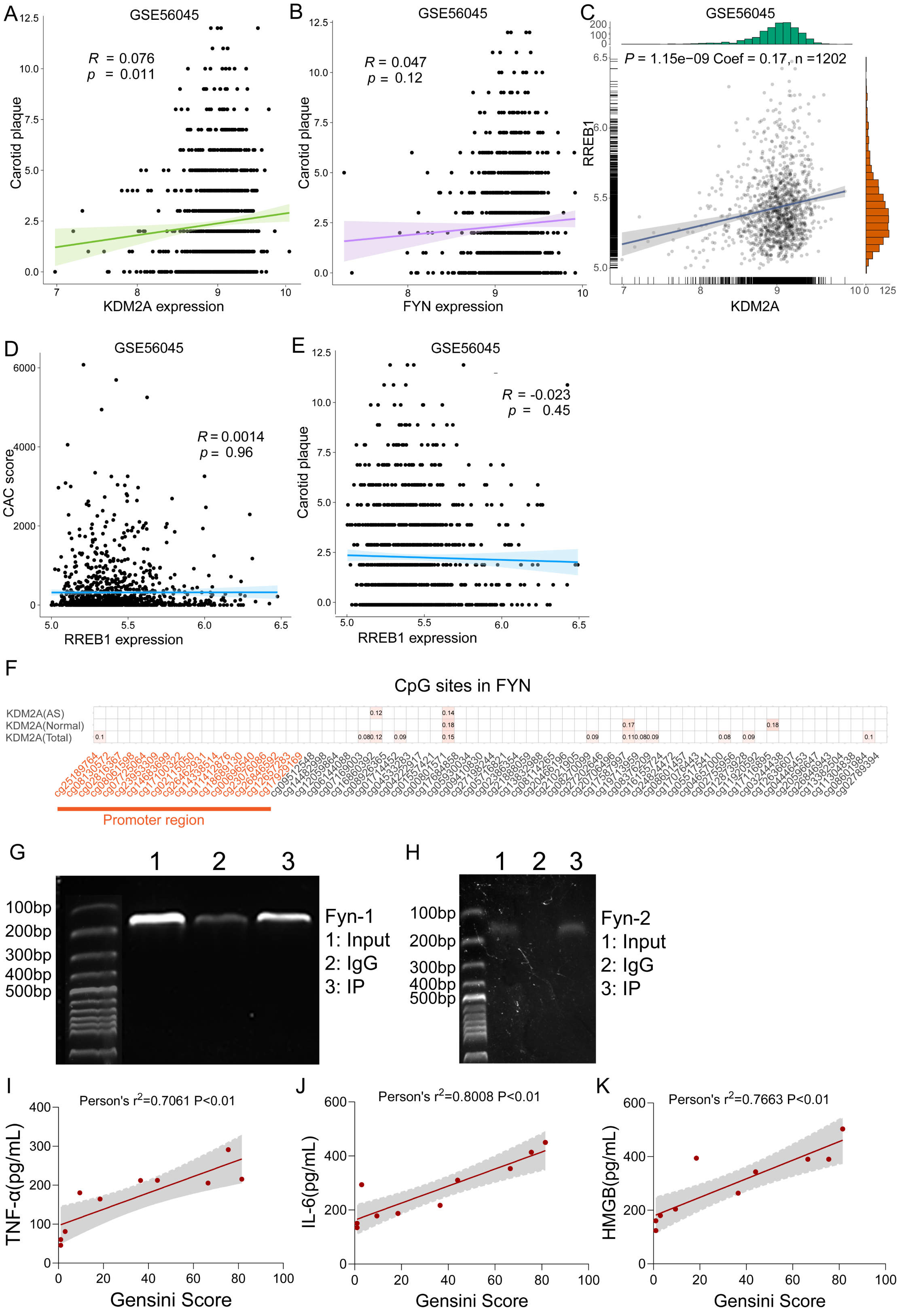

A
GSE56045
R = 0.076
p = 0.011
Carotid plaque
KDM2A expression
B
GSE56045
R = 0.047
p = 0.12
Carotid plaque
FYN expression
C
GSE56045
P = 1.15e−09 Coef = 0.17, n =1202
RREB1
KDM2A
D
GSE56045
R = 0.0014
p = 0.96
CAC score
RREB1 expression
E
GSE56045
R = -0.023
p = 0.45
Carotid plaque
RREB1 expression
F
CpG sites in FYN
KDM2A(AS)
KDM2A(Normal)
KDM2A(Total)
Promoter region
G
1 2 3
100bp
200bp
300bp
400bp
500bp
Fyn-1
1: Input
2: IgG
3: IP
H
1 2 3
100bp
200bp
300bp
400bp
500bp
Fyn-2
1: Input
2: IgG
3: IP
I
Person's r2=0.7061 P<0.01
TNF-α(pg/mL)
Gensini Score
J
Person's r2=0.8008 P<0.01
IL-6(pg/mL)
Gensini Score
K
Person's r2=0.7663 P<0.01
HMGB(pg/mL)
Gensini Score

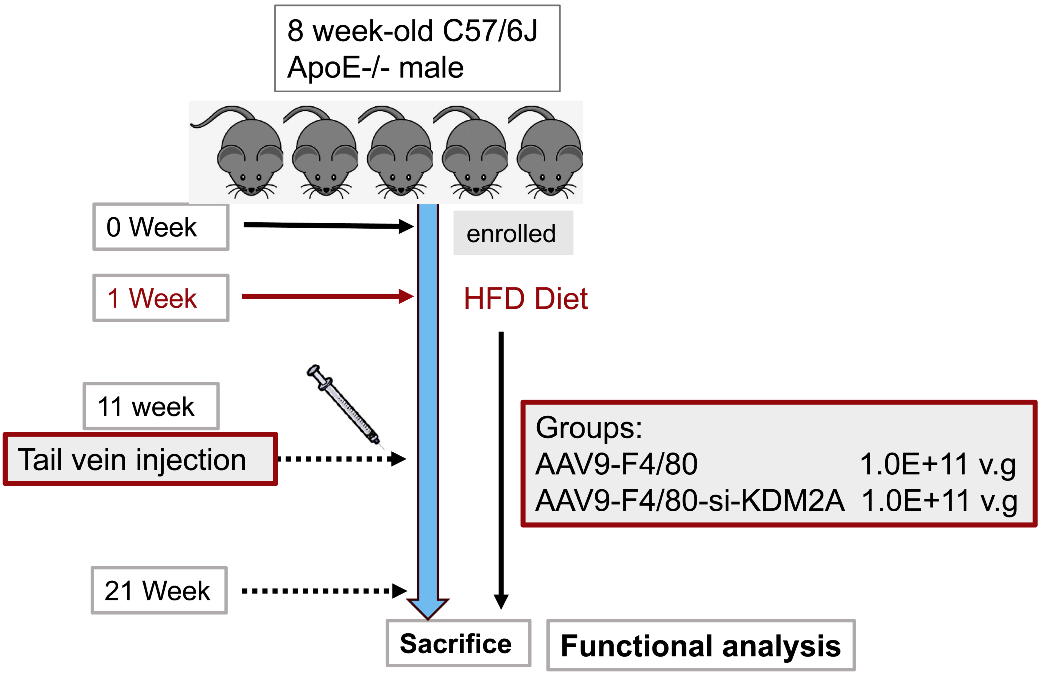
8 week-old C57/6J
ApoE-/- male
0 Week
enrolled
1 Week
HFD Diet
11 week
Tail vein injection
Groups:
AAV9-F4/80 1.0E+11 v.g
AAV9-F4/80-si-KDM2A 1.0E+11 v.g
21 Week
Sacrifice
Functional analysis

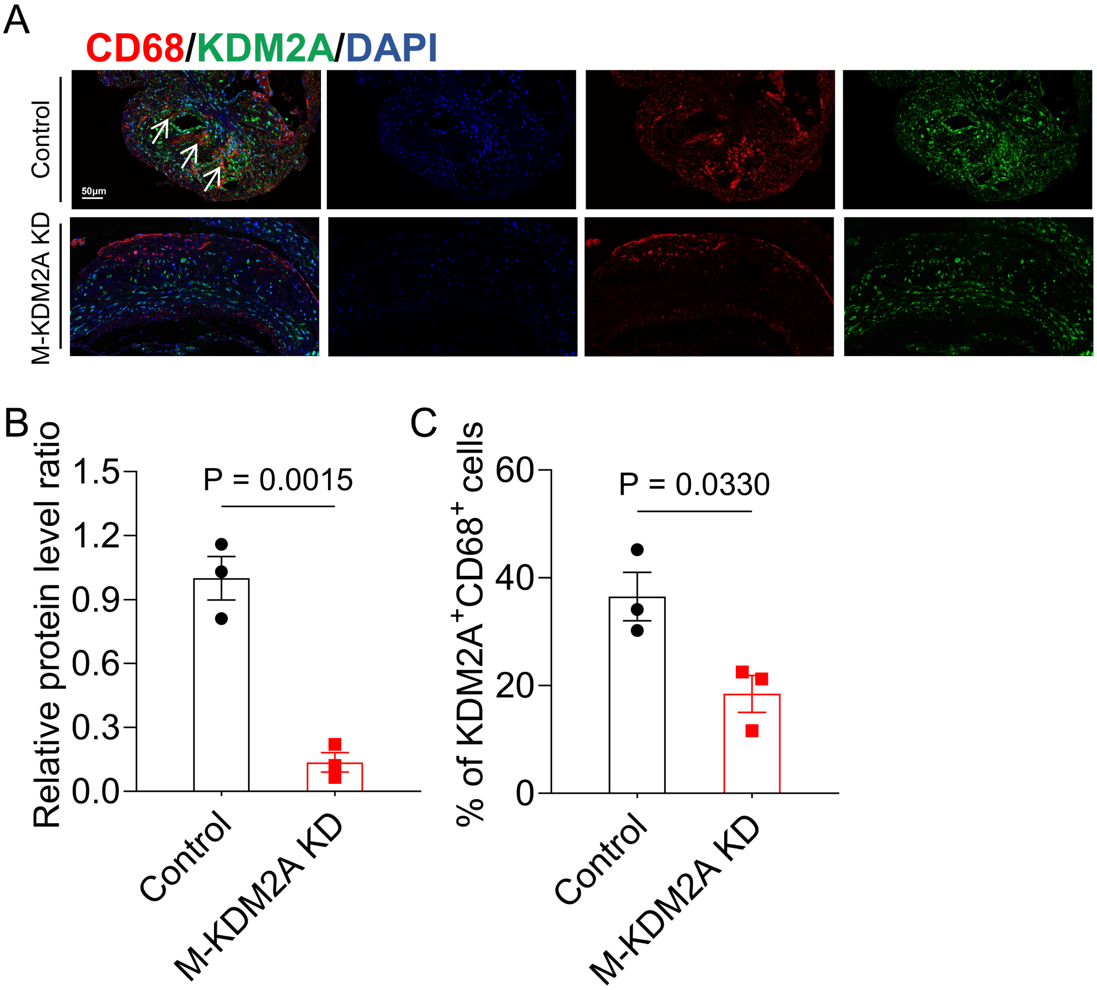
A
CD68/KDM2A/DAPI
Control
M-KDM2A KD
50μm
B
Relative protein level ratio
1.5
1.2
0.9
0.6
0.3
0.0
P = 0.0015
Control
M-KDM2A KD
C
% of KDM2A⁺CD68⁺ cells
60
40
20
0
P = 0.0330
Control
M-KDM2A KD

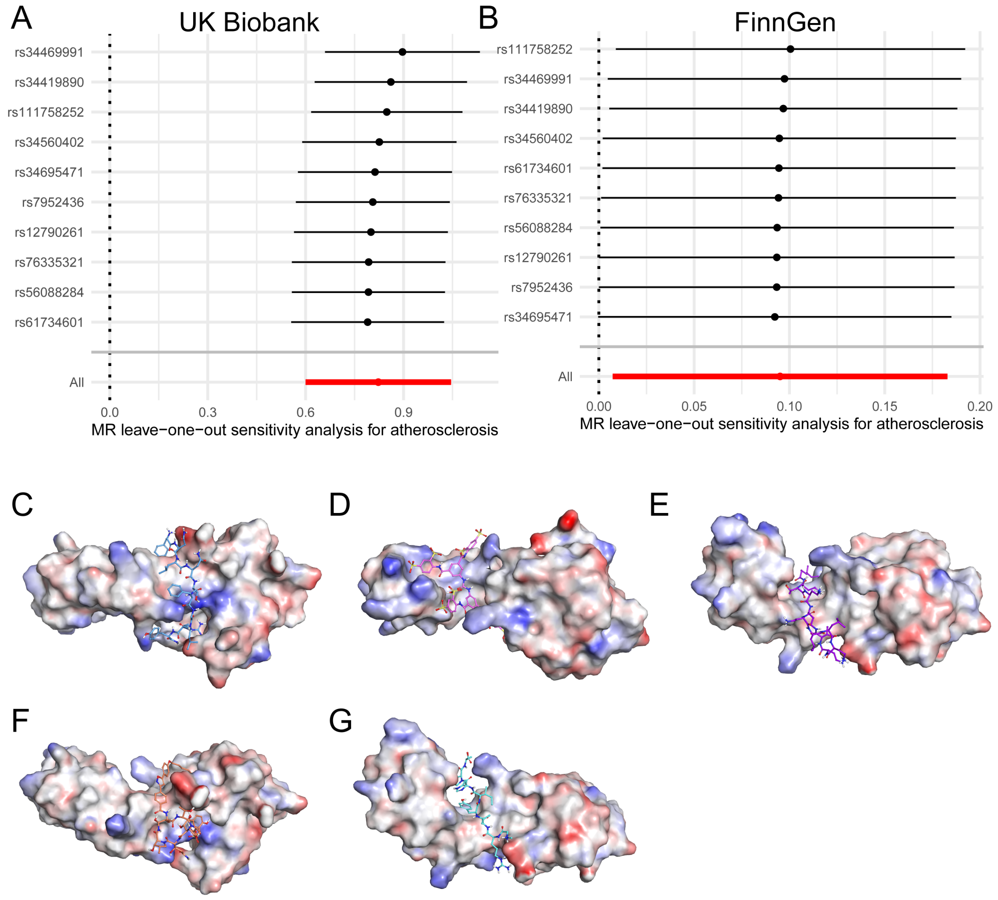
A
UK Biobank
rs34469991
rs34419890
rs111758252
rs34560402
rs34695471
rs7952436
rs12790261
rs76335321
rs56088284
rs61734601
All
0.0
0.3
0.6
0.9
MR leave−one−out sensitivity analysis for atherosclerosis
B
FinnGen
rs111758252
rs34469991
rs34419890
rs34560402
rs61734601
rs76335321
rs56088284
rs12790261
rs7952436
rs34695471
All
0.00
0.05
0.10
0.15
0.20
MR leave−one−out sensitivity analysis for atherosclerosis
C
D
E
F
G
